# Network inequality through preferential attachment, triadic closure, and homophily

**DOI:** 10.1038/s41598-026-42911-3

**Published:** 2026-03-13

**Authors:** Jan Bachmann, Samuel Martin-Gutierrez, Lisette Espín-Noboa, Nicola Cinardi, Fariba Karimi

**Affiliations:** 1 Complexity Science Hub, 1030 Vienna, Austria; 2 Department of Network and Data Science, Central European University, 1100 Vienna, Austria; 3 Graz University of Technology, 8010 Graz, Austria; 4 Grupo de Sistemas Complejos, ETS de Arquitectura de Madrid, Universidad Politécnica de Madrid, 28040 Madrid, Spain; 5 Faculty of Engineering, Free University of Bozen-Bolzano, 39100 Bolzano, Italy; 6 Center for Computational and Stochastic Mathematics, Instituto Superior Tecnico, Universidade de Lisboa, 1049-001 Lisbon, Portugal

**Keywords:** Mathematics and computing, Physics

## Abstract

Inequalities in social networks arise from linking mechanisms, such as preferential attachment (connecting to popular nodes), homophily (connecting to similar others), and triadic closure (connecting through mutual contacts). Preferential attachment drives degree inequality and homophily drives segregation, but we know less about how these two mechanisms interact with triadic closure. This gap limits our understanding of how network inequalities emerge. We introduce PATCH, a network growth model that combines all three mechanisms, and use it to study how they create disparities within and between two groups in undirected networks. Simulations show that homophily and preferential attachment increase segregation and degree inequality. Triadic closure has varied effects: conditional on the other mechanisms, it increases population-wide degree inequality while reducing segregation and between-group degree disparities. We demonstrate PATCH’s explanatory potential using fifty years of Physics and Computer Science collaboration and citation networks exhibiting persistent gender disparities. PATCH reproduces these gender disparities when it combines preferential attachment, moderate gender homophily, and triadic closure. By connecting mechanisms to observed inequalities, PATCH shows how their interplay sustains group disparities and how improving one inequality dimension may affect others.

## Introduction

The distribution of visibility in social networks is typically highly unequal, with a small fraction of individuals accumulating a disproportionate number of links ^1,2^. This inequality can have far-reaching consequences, as network visibility can determine access to social capital, information, and opportunities ^3,4^. At the population level, the accumulation of links manifests as *degree inequality*, where visibility concentrates among a few highly connected individuals. Within social groups, such concentration can persist as *within-group inequality*, so that visibility accrues mainly to a small set of individuals. This can be a consequence of tokenistic inclusion ^5^, where only a few members of the minority are included to meet required quotas while most remain peripheral ^6^. Under *segregation*, ties cluster within groups and cross-group interaction becomes rare, potentially restricting information flow and support and distorting perceptions of group sizes ^7^, increasing economic inequalities downstream ^8,9^. Finally, networks can exhibit *between-group degree inequalities*, where one group is systematically less visible and thus less likely to be reached, cited, or recognized, leading to further cumulative disadvantages. Women, a minority group in scientific fields like Physics ^10,11^, face such challenges in accessing and distributing information due to their reduced visibility relative to men in networks of scientific collaboration ^12,13^.

Inequalities in network structure can arise from biased linking decisions. People’s tendency to form social ties with those who already have many connections, known as *preferential attachment*, is one of the mechanisms that can lead to unequal degree distributions ^14^. While preferential attachment is a driver of inequality in many social systems, such as academic collaboration ^15,16^, these networks also exhibit a high degree of *triadic closure* ^17,18^. As a tendency for individuals to connect to friends-of-friends, triadic closure reinforces local clustering in social networks and affects network segregation ^19^. A third important mechanism is *homophily*, the tendency to form ties with similar others ^20^, which can lead to visibility inequalities through segregation ^21^.

These mechanisms interact. Instead of an explicit bias towards popularity, preferential attachment can also be an implicit consequence of triadic closure, since well-connected individuals are more likely to appear among friends-of-friends ^22^. In combination with preferential attachment, homophily can push one group to the periphery of the network ^23^, create false perceptions of group sizes ^7^ or disadvantage a minority in terms of their connectivity ^21,24^. Whether triadic closure increases or decreases segregation depends on whether homophily also shapes the selection among friends-of-friends ^19,25^. This could further isolate a minority group and thus restrict their access to valuable information ^12,26^ or exacerbate health disparities ^27^. The interaction of mechanisms matter as the effect of one mechanism can change when another is present ^19,25^.

By creating diverse synthetic networks, mechanistic models can be used to study the impact of biased link formation mechanisms on network inequality ^21,24^ and interventions ^28^. While existing models have been used to study interacting mechanisms ^14,19,21,22,25,26,29,30^, they have not included all three or analyzed only single dimensions of inequality in the presence of a minority group. We introduce PA–TC–H, a network growth model extending a branch of models originating from the preferential attachment (PA) Barabási-Albert model ^14^ by combining existing triadic closure (TC) ^29,30^ and homophily (H) ^21^ extensions.

Our comprehensive analysis shows that homophily and preferential attachment can segregate a minority from the majority group in the network, and increase degree inequality within each group and among all nodes. Homophily also increases the degree disparity between the minority and majority groups. Triadic closure mitigates segregation and between-group inequality only when the selection among friends of friends is not biased by the other two mechanisms. To identify the mechanisms and parameters that best explain the varying gender inequalities in scientific collaboration and citation, we apply likelihood-free inference to three empirical networks. We find that the empirically observed inequalities are best explained by a model where both global search and local triadic closure links are biased by preferential attachment and homophily. Our results suggest that mitigating inequalities based on link formation can only be achieved when keeping all mechanisms and other inequalities in mind, as isolating one can lead to unintended consequences.

## Results

**Fig. 1:**
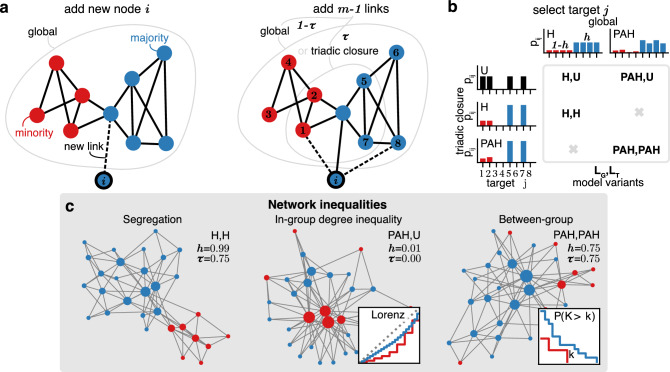
PATCH networks. (**a**) Network growth: A new node *i* joins the network and is assigned to the majority group with probability $1 - f_{\min }$, or the minority otherwise. For its first link, *i* selects a target node globally, from all existing nodes (gray outline). Subsequently, *i* creates $m-1$ additional links. For each new link, *i* connects to a target either globally with probability $1-\tau$, as in the first link, or through triadic closure, limiting target nodes to friends of friends $j \in \{1, 2, 5, 7\}$ (inner gray outline). (**b**) Linking mechanisms: Among the available triadic closure target nodes *j*, *i* chooses based on one of three mechanisms $p_{ij}$: Targets are chosen uniformly at random without any bias ($\textrm{U}$), solely based on group membership with homophily ($\textrm{H}$), or by combination of degree-based preferential attachment and homophily ($\textrm{PAH}$). While the mechanisms may vary between global and triadic closure links, we consider only model variants $\mathrm {(L_G,L_T)}$ in which triadic closure is unbiased ($\mathrm {L_T} {:=}\textrm{U}$) or biased in the same way as the global selection $\mathrm {L_T} {:=}\mathrm {L_G} \in \{ \textrm{H}, \textrm{PAH} \}$. Homophily is controlled by a parameter *h* favoring in-group links for $h>0.5$ or out-group links for $h<0.5$. (**c**) Network inequalities: Three PATCH networks with high segregation, unequal degrees, and with one group dominating the other, illustrating the three network outcomes we analyze.

PATCH is based on the growth mechanism of the Barabási-Albert network model which sequentially adds a total of *N* nodes to the network, each linking to *m* previously added nodes (Fig. 1)^14^.  Following existing triadic closure variants of this model, a probability $\tau$ determines whether the pool of potential target nodes for each new link is restricted to friends-of-friends or includes all nodes in the existing network^29,30^.  We refer to the former as triadic closure links and the latter as global links. To choose among the target nodes, we consider three different mechanisms: unbiased selection ($\textrm{U}$), homophily ($\textrm{H}$), and preferential attachment with homophily ($\textrm{PAH}$). Following another common extension, we control homophily by a parameter $h \in (0,1)$ with a preference for in-group links for $h > 0.5$ and out-group connections for $h < 0.5$^21^.  To model social group dynamics, we assume each node belongs to either a minority or majority group, with the minority group representing a fraction $f_{\min }< 0.5$ of the *N* nodes. In the presence of preferential attachment ($\textrm{PAH}$), the probability of forming a link is proportional to the degree of the target node, matching the baseline model^14^.  The formal definitions and parameterizations of all mechanisms are detailed in “Methods”. Since prior literature suggests a reduced importance of homophily when choosing triadic closure targets^31^,  we vary mechanisms between global and triadic closure links. To this end, we distinguish four global ($\mathrm {L_G}$) and triadic closure ($\mathrm {L_T}$) link mechanism combinations $\mathrm {(\mathrm {L_G},\mathrm {L_T})} \in \{\mathrm {(H,U)},\mathrm {(H,H)},\mathrm {(PAH,U)},\mathrm {(PAH,PAH)}\}$. We then simulate the model with $N=5\,000$ nodes and $m=3$ initial links, and a minority fraction of $f_{\min }= 0.2$ while varying *h* and $\tau$ as control parameters.

### Network segregation

**Fig. 2:**
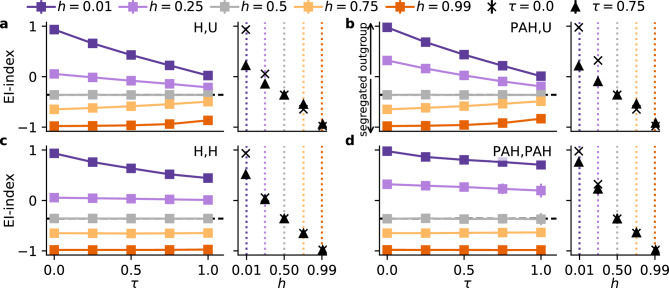
Homophily drives segregation. We vary the triadic closure probability $\tau$ and homophily *h* (x-axes of the wider and shorter subplots, respectively) and the link formation combinations (**a**–**d**) to measure the network segregation by the EI-index (y-axis). Negative values indicate segregation, neutral values indicate mixing, and positive values indicate outgroup linking. (**a**) Due to the group size imbalance, the network is slightly segregated ($\textrm{EI} = -0.36$) in the neutral case ($h=0.5$ and $\tau =0.0$; see Section S2). (**a**–**d**) Segregation is driven by homophily *h* (from purple to orange). Under homophilic preferences ($h > 0.5$) the network becomes segregated, while heterophilic tendencies ($h < 0.5$) lead to a high inter-group connectivity. The effect of triadic closure is not straightforward. (**a**,**b**) If the selection is unbiased ($\mathrm {L_T} = \textrm{U}$), it moderates strong segregation or strong mixing towards the neutral state (gray color). (**c**,**d**) If the selection is biased ($\mathrm {L_T} = \mathrm {L_G}$) and nodes prefer heterophilic linking ($h < 0.5$), triadic closure slightly shifts the network towards more in-group linking. Under homophily ($h > 0.5$), triadic closure has no effect.

*Network segregation* can isolate a group from accessing information, opportunities or support, thus driving socio-economic inequalities^9^.  The observed mixing preferences between various groups in a network depend not only on the homophily preference of individual actors, but also on the local network structure that they are embedded in, the presence of other link formation mechanisms ^25,32^, or opportunity constraints such as residential segregation^8^.  The effect of triadic closure on network segregation is not straightforward. While some studies suggest that triadic closure can moderate network segregation^19^ , others argue that it can amplify it^25^.  The main distinction is the presence of homophily in the selection of triadic closure targets, that is, whether nodes prefer to link to friends of friends with similar attributes^19^.  Empirical studies suggest that the effect of homophily, while still present, decreases with the proximity of target nodes^31^.  In combination, this suggests that triadic closure can moderate segregation. We investigate the effect of triadic closure on network segregation with or without a biased selection of triadic closure targets by choosing $\mathrm {L_T}=\mathrm {L_G}$ or $\mathrm {L_T}=\textrm{U}$, respectively.

We measure the segregation of the simulated network by the fraction of the frequency of internal links *I* to external links *E* as

1 $$\begin{aligned} EI = \frac{E - I}{E + I} \end{aligned}$$

ranging from $-1$ for fully segregated networks to $+1$ for networks in which members of one group exclusively link to the other group (Fig. 2)^33^.  EI captures whether cross-group contact is common or rare, independent of how many ties individuals have. Varying the homophily $h\in \{0.01, 0.25, 0.50, 0.75, 0.99\}$ and the triadic closure probability $\tau \in \{0.0, 0.25, 0.5, 0.75, 1.0\}$, we identify the strongest effect with increasing *h*. Homophily drives network segregation, regardless of any combination of link formation mechanisms.

We observe a moderating effect of triadic closure, driving the system towards a slightly segregated state which aligns with the neutral case (Fig. 2a with $h=0.5$; see Section S2 for a derivation of the neutral $\textrm{EI}$-index). Surprisingly, triadic closure does not seem to amplify segregation, even when it is biased itself ($\mathrm {(H,H)}$ and $\mathrm {(PAH,PAH)}$, bottom row). There is no amplification of segregation in the network, as the EI-index remains constant over increasing $\tau$. Only in the case of heterophily ($h < 0.5$, purple coloring) we observe a decrease in the EI-index, indicating that triadic closure counteracts cross-group mixing by favoring within-group ties. This is surprising, given that triadic closure, too, is biased towards heterophily. Taken together, these results clarify when triadic closure amplifies or moderates segregation: (i) in agreement with existing studies^19^,  triadic closure moderates segregation when it is unbiased. (ii) It does not amplify segregation under homophily ($h>0.5$) and this is not explained by the presence of preferential attachment (see $\mathrm {(H,H)}$ in Fig. 2). (iii) In the heterophilic case ($h<0.5$), it moderates the abundance of outgroup links towards a well-mixed network, regardless of whether it is biased or not.

### Degree inequality

**Fig. 3:**
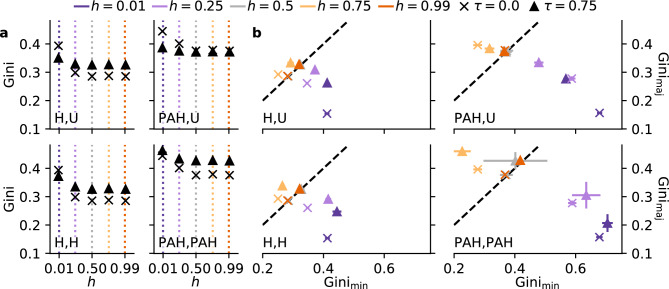
Preferential attachment drives degree inequality. (**a**) Measuring global degree inequality by the Gini coefficient, we identify the strongest effect by the presence of preferential attachment $\mathrm {(PAH,U)}$ and $\mathrm {(PAH,PAH)}$. In heterophily ($h < 0.5$), only a few nodes receive most of the links, even in the absence of preferential attachment. While the effect of triadic closure depends on the other mechanisms, it exacerbates degree inequality in most cases. (**b**) In-group degree inequality behaves similarly. Degree inequality is higher for the majority group under homophily. While extreme homophily equalizes the difference between inequalities, the in-group inequality is strongest for the minority under strong heterophily.

Social networks tend to exhibit strong *degree inequalities* as the number of links individual nodes accumulate is distributed unevenly^1^.  The Gini coefficient is a common measure to quantify this inequality, ranging from zero for a perfectly equal distribution towards one for a network in which a single node centers all links^24^.  Through their increased visibility, such hub nodes can disproportionately access and distribute information, opportunities, and social capital^3,4^.  Prior literature has identified preferential attachment^14^,  especially in combination with extreme heterophily or homophily ^24^ as a key driver of degree inequality in social networks. At the same time, triadic closure can act as sub-linear preferential attachment (i.e., the connection probability is $p_{ij} \propto k_j^\alpha$ with $\alpha < 1$)^22^,  potentially exacerbating degree inequality.

The Gini coefficient is mostly driven by the presence of preferential attachment in our model (Fig. 3a). In the absence of preferential attachment and triadic closure (left column and $\tau =0$), the Gini coefficient generally decreases with homophily *h*, as nodes tend to link more and more to similar others. This occurs because in the heterophilic setting ($h < 0.5$), a few minority nodes receive most links from the majority group^21^.  Our results show that this is true even in the absence of preferential attachment; an increase in homophily would motivate minority nodes to link to other minority nodes, decreasing the skewness of the distribution.

Apart from the extreme heterophily scenario, triadic closure exacerbates degree inequality, which is in line with the sub-linear preferential attachment it creates^22^.  This is due to a degree-biased sampling as high-degree nodes are more likely to appear among friends of existing neighbors^22,30^.  To distinguish the two types of preferential attachment, we define the explicitly modelled preference to connect to popular nodes as *choice preferential attachment* and the effect of triadic closure as *induced preferential attachment*.

When nodes globally choose based on choice preferential attachment, but are locally unbiased $\mathrm {(PAH,U)}$, or under extreme heterophilic preference ($h=0.01$) without any choice preferential attachment ($\mathrm {(H,U)}$ and $\mathrm {(H,H)}$), the Gini coefficient decreases with $\tau$ (Fig. 3a). In the former case, triadic closure reduces the number of links drawn based on preferential attachment. Compared to choice preferential attachment, the induced preferential attachment is sub-linear, which then reduces the overall degree inequality. In the second scenario, triadic closure likely reduces degree inequality by shifting links towards the larger majority group (compare reduced segregation in Fig. 2a, c), thereby spreading links across more individuals.

In the presence of two groups of unequal size, global degree inequality can stem from asymmetric inequalities within each group. Measuring the inequality per group by the Gini coefficient of the degree distribution of the minority $\mathrm {Gini_{min}}$ and majority group $\mathrm {Gini_{maj}}$ separately, we observe a negative correlation between the two coefficients (Fig. 3b). If one group has a high degree inequality, the other group typically has a more evenly distributed degree visibility. This further indicates that the global degree inequality is mainly driven by the more unequal group. Namely, the degree distribution of the majority group is more unequal under intermediate homophilic preferences ($h = 0.75$), while the minority group is more unequal with heterophilic mixing ($h < 0.5$). At the same time, the other group shows less inequality than the neutral case. This is especially pronounced when both global and triadic closure links are biased by preferential attachment and homophily $\mathrm {(PAH,PAH)}$. While one group shows the most extreme inequality, the other one remains almost equal. Only in the absence of homophily ($h=0.5$) or extreme homophily ($h=0.99$), we observe identical, but high Gini coefficients for both groups and all model variants.

### Between-group degree inequality

**Fig. 4:**
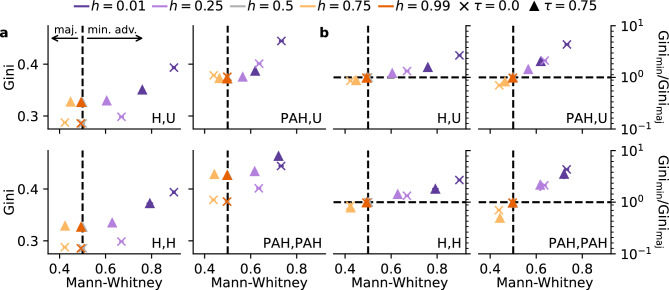
In- and between-group inequality. (**a**) We measure between-group inequality, the capability of one group to accumulate more links than the other, by the Mann-Whitney statistic. Values below 0.5 indicate that the majority group are advantaged in degree visibility, while values above 0.5 indicate the opposite. Homophily drives between-group inequality while unbiased triadic closure and the presence of preferential attachment moderate it. We reproduce in our undirected network model the U-shaped relation between all-node and between-group degree inequality found in a similar, directed model^24^.  One group being more visible than the other always comes with a higher degree inequality among all nodes. (**b**) This inequality is always higher for the advantaged group. In the heterophilic case ($h < 0.5$), the minority group is disadvantaged and shows a higher inequality. Under intermediate homophily levels ($h = 0.75$), the advantage of the majority group matches their increased degree inequality. This indicates that an advantage in degree visibility favors only a few nodes from one group. Only unbiased triadic closure consistently neutralizes this effect.

Degree inequality, whether measured in the population or within groups, is agnostic about whether one group is advantaged over the other. We therefore consider *between-group degree inequality*, capturing whether degree visibility tends to be systematically higher in one group than the other. Prior literature has identified homophily as a key driver of between-group inequality^21,24^. As a potential moderator or amplifier of homophily, the effect of triadic closure on between-group inequality remains unclear. We measure the between-group inequality between the two groups as the probability that a randomly chosen node from the minority group has a higher degree than a randomly chosen node from the majority group. This measure reflects the common language interpretation ^34^ of the Mann-Whitney test statistic ($\textrm{MW}$)^35^.  Values above $50\%$ indicate that the minority group has a higher degree visibility than the majority group, while values below $50\%$ indicate the opposite. Because it is rank-based, $\textrm{MW}$ reflects distributional dominance across the full degree distributions rather than differences in means.

In agreement with previous works^21,24^,  we identify homophily as the main driver of between-group inequality (changes in the x-axis of Fig. 4). In the intermediate homophily case ($h = 0.75$), the majority group achieves greater visibility than the minority group. The opposite is true under heterophily ($h < 0.5$), when the minority group dominates the majority. Triadic closure and preferential attachment have a moderating effect on between-group inequality instead, as they reduce it towards the neutral value of $50\%$.

The relationship between between-group inequality and all-node degree inequality is U-shaped, as previously observed in a similar, but directed network model^24^.  Whenever one group is more visible than the other, the degree inequality among all nodes is higher. This occurs irrespective of the presence of preferential attachment or triadic closure and it is strongest in the heterophilic case ($h < 0.5$), where the minority group is advantaged. In Fig. 4b, we link between-group inequality to the intra-group inequality summarized by the ratio of the minority and majority groups’ Gini coefficients $\mathrm {Gini_{min}/Gini_{maj}}$. We observe a positive linear trend between between-group inequality and the intra-group inequality in all model variants, confirming that the advantaged group always has a higher degree inequality. The presence of preferential attachment shifts this effect away towards lower between-group inequality but higher in-group inequality among the advantaged group. The advantage in degree visibility favors only a few nodes from the dominant group while most nodes remain less visible. While nodes in the disadvantaged group have a more even degree distribution, they remain less visible overall.

**Table 1:** Qualitative summary of outcomes. Linking mechanisms (PA, TC, H) affect network segregation (EI), in-group (Gini) and between-group (MW) degree inequality. Single triangles and color indicate high or low metric values and double triangles symbolize neutralizing changes towards equality. Effects may be general, or depend on preferential attachment (‘PA’), heterophily (‘het’), or homophily (‘hom’). Color shades indicate the effect strength, with the darkest shade marking the mechanism that contributes the strongest to the respective inequality. PA does not affect segregation or between-group inequality, but it skews the degree distribution of minorities, and of majorities only under homophily. The effects of triadic closure depend on whether it is biased (node choice follows the global mechanism,$\mathrm {L_T}=\mathrm {L_G}$) or unbiased (uniform choice,$\mathrm {L_T} = \textrm{U}$). In the unbiased case, triadic closure reduces segregation and between-group inequality. In the biased case, it neutralizes segregation only in heterophilic networks. Homophily segregates networks under homophilic preferences and towards out-group linking under heterophily. Moving from its absence to heterophily or to moderate homophily creates between-group inequality, favoring the minority or majority group, respectively. The favored group experiences higher degree inequality, indicating that only a few nodes benefit from the advantage.

Mechanism	Parameterization	Segregation (EI)	In-group inequ. (Gini)			Between-group (MW)
			All	Min	Maj	
Preferential attachment	set $\mathrm L_{G,T}$ to $\textrm{PAH}$	–			^hom^	–
Triadic closure	unbiased, increase $\tau$		_PA_	–	–	
	biased, increase $\tau$	_het_	–	–		–
Homophily	Towards heterophily					
	Moderate homophily		–			
	Extreme homophily					

– No or mixed effect   Metric increase   Decrease   Neutralization   Main up- or downwards driver/neutralizer

PATCH produces a wide variety of networks and inequalities by variation of its mechanisms. The simulation results are robust to changes in network size *N*, minority fraction $f_{\min }$, and number of new links per node *m* (Figures S1 to S5 and S7 to S16). Table 1 summarizes the main effects and interplays of the three mechanisms on segregation, in-group and between-group inequality. Given an observed network, the table maps its inequalities to contributing mechanisms, providing explanations and guiding the implementation of mitigation strategies.

### Network inequality in academic networks

**Fig. 5:**
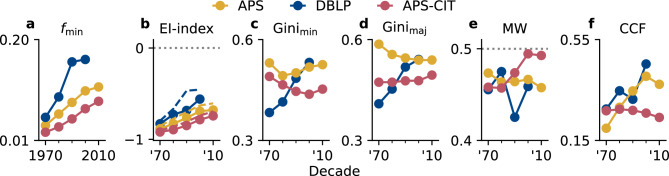
Gender inequalities in scientific networks over five decades. Each subplot shows an aggregate network statistic summarizing observed network inequalities and informing the PATCH model parameter inference. (**a**) While the fraction of women as the minority group $f_{\min }$ is increasing consistently over time for all datasets, it remains well below parity. (**b**) The observed segregation evolves in line with our expectation (dashed lines; see Section S2) based on the growing $f_{\min }$. Still, the networks remain slightly more segregated than this baseline suggests. (**c**,**d**) Within-group inequality and how it is distributed among men and women varies by dataset and time. APS collaboration exhibits the strongest degree inequality in the 1970s, decreasing consistently only among men. Degree inequality in APS citations shows a reversed trend instead. Collaboration among DBLP authors is quickly becoming more unequal in the entire population. (**e**) Despite their growing representation women are disadvantaged in all datasets and decades except in citations starting in the 1990s, as measured by the Mann-Whitney statistic (MW). (**f**) The average clustering coefficient (CCF), measuring the fraction of connected neighbors out of all possible pairs of neighbors, increases in collaboration and decreases in citation.

We apply PATCH to three empirical networks, considering scientific collaborations within journals of the American Physical Society (APS)^36^,  citations among APS publications (APS-CIT) ^36^ and scientific collaborations within the Computer Science Bibliography (DBLP)^37^.  In collaboration networks (APS and DBLP), scientists are nodes connected by joint publications. In line with previous works on gender inequalities in citation networks, we define publications as nodes, with gender assigned by the first author for APS-CIT^38^.  Links are created if one publication cites another one (see “Methods” for details on the network creation process and Table S1 for summary statistics). These datasets reflect the network of collaborations and citation practices among researchers in the fields of Physics and Computer Science, respectively; fields in which women are historically underrepresented^10,39^.  Collaboration networks are moderately segregated by gender ^32,40,41^ and highly clustered^18^,  suggesting the presence of triadic closure and homophily in the decision-making process of forming collaborations. The number of collaborators per author is highly unequal, with a few authors having many collaborators and most authors having only a few collaborators ^18^ which can be attributed both to preferential attachment and triadic closure^16^.  Identifying the presence of biases, such as preferential attachment, gender homophily or triadic closure in the formation of collaborations is crucial to reduce the inherent inequalities in science in general ^15^ and between men and women specifically^10,42^.  With whom an author gets to collaborate can greatly influence their visibility and career prospects^43–46^.  As accumulated citations are often used to measure scientists’ impact, gender homophilic citation practices ^38^ could drive the inequalities in citations received^10,47^.  Although PATCH is generally agnostic to the context it is applied to, incorporating preferential attachment, triadic closure, and (gender) homophily makes it a valuable tool to understand the role of latent behavioral biases on the emergence of inequalities.

Considering a roughly equal representation of men and women in the underlying general population, the fraction of women in Physics and Computer Science increased only slowly between 1970 and 2010 (Fig. 5a). Even DBLP, which shows the highest increase, remains well below 20% of women in the 2000s. The low fraction of women-led publications $f_{\min }$in the citation network is likely linked to women’s higher dropout rates^48^,  shorter careers^10^,  and unequal parental obligations^49^.  As women’s representation increases, we observe an upwards trend in across-gender collaborations and citations over time as indicated by an increasing $\textrm{EI}$-index (Fig. 5b). While the trend matches our expectation (see Section S2 for a derivation of the neutral $\textrm{EI}$-index), the observed networks are slightly more segregated. Given our simulation results, this observation suggests the presence of gender homophily.

While men and women share a strong degree inequality, the three datasets show distinctive trend variations (Fig. 5c, d). In the citation network, inequality levels appear to decrease for women, but increase for men. In contrast, APS shows the reversed effect and degree inequalities in DBLP sharply increase for both groups. Considering the between-gender inequality, we observe persistently lower degrees for women in terms of number of co-authors and citations in line with prior research (Fig. 5e)^10^.  Only the citation network develops towards the neutral state in which men and women share the same degree visibility. Note however that all measures are computed irrespective of the link direction and thus incorporate both outgoing and incoming citations.

Besides the presented network inequality statistics, we also consider the average local clustering coefficient (CCF) of the network to better capture the effect of triadic closure. The observed CCF is generally higher for the collaboration networks following the expected prevalence of triadic closure, ^18^ but also the construction of the network itself (Fig. 5f). Pair-wise connections among all co-authors of a paper form many triangles among them, leading to high CCF values. Trends are increasing for the collaboration networks, but decreasing for citations.

### Underlying behavioral mechanisms

**Fig. 6:**
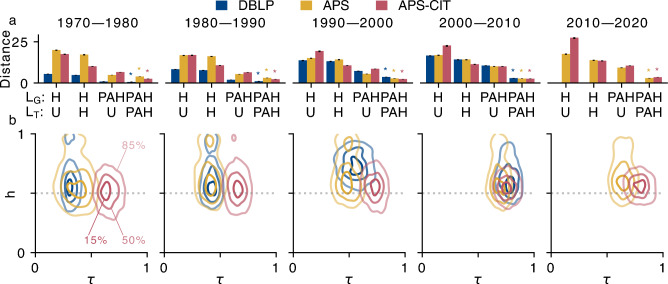
Likelihood-free inference of PATCH variant and parameters. (**a**) Euclidean distance between observed and simulated network statistics for the empirical networks (color) and the PATCH model variants over decades. The best model variant is chosen based on the minimum distance (marked by a star; see “Methods”). $\mathrm {(PAH,PAH)}$ performs best in all cases, indicating that both the global and triadic closure target selection is biased by popularity and gender-homophily. (**b**) Approximate joint posterior distributions of the homophily parameter *h* and triadic closure probability $\tau$ for $\mathrm {(PAH,PAH)}$ over time with the contours incorporating $85\%$, $50\%$ and $15\%$ of the posterior samples. The distributions show a moderate gender-homophily and triadic closure preferences.

To estimate which behavioral biases could explain the observed inequalities in the empirical networks, we apply Approximate Bayesian Computation (ABC) to estimate the posterior parameter distributions of *h* and $\tau$ that best explain the observed network statistics^50^.  ABC uses the distance between summary statistics of the observed and simulated networks to sample from an approximate posterior distribution of the model parameters. We choose the inequality metrics and average clustering coefficients as summary statistics, fix the network size *N*, compute $f_{\min }$and *m* directly from the data and fit only *h* and $\tau$ (see “Methods” for details).

After validating the inference approach on simulated data (see Figs. S17 to S21 and “Methods” for details), we apply it to the empirical networks. We choose the model variant of PATCH that minimizes the Euclidean distance between the observed and simulated network statistics (Fig. 6.**a**). There is strong variation in how well model variants fit the observed inequalities. Over time, the second best model variant $\mathrm {(PAH,U)}$ becomes less viable in all datasets, indicating that triadic closure selection is increasingly likely to be biased by homophily and preferential attachment. In all datasets and decades, the $\mathrm {(PAH,PAH)}$ variant best reproduces the observed network statistics and inequalities. In contrast to existing studies^31^,  popularity and similarity also seem to shape linking selection among local authors.

Samples of the approximate posterior distributions of parameters *h* and $\tau$ for the $\mathrm {(PAH,PAH)}$ models estimate the latent behavior that best explains the observed inequalities (Fig. 6**b**, see also “Methods” and Figs. S22 to S27). Both collaborations and citations in most decades are mildly gender-homophilic and show moderate triadic closure tendencies that increase slightly over time across all networks. This suggests that scientists increasingly base their collaboration and citation choices on friends-of-friends, and that these choices depend on author popularity and gender (implicitly or explicitly). Adding to the ongoing discussion on gender biases in citation practices^38,47^,  this suggests that biases are present both in the global and local selection of citations.

Our inference model reproduces the high levels of segregation, the within-group inequality ratio, and between-gender inequality in the empirical networks (Figs. S22 to S25 and S27). Linking this to our simulation results for $\mathrm {(PAH,PAH)}$ (Figs. 2, 3, 4), this suggests that reducing gender homophily could improve women’s segregation and degree disadvantage. At the same time, this would decrease the degree inequality among men, at the expense of an increase for women, suggesting that only a few, already popular women would benefit from the majority group’s attention shift. This trade-off stabilizes the global degree inequality which can only be reduced by decreasing the effect of preferential attachment or triadic closure. To move the empirical networks towards a more equal state in all inequality dimensions, a simultaneous reduction of all mechanisms is required.

## Discussion

PATCH combines the common link formation mechanisms of preferential attachment, homophily, and triadic closure, and shows how they jointly shape network inequality. We find that the presence of unbiased triadic closure can have a moderating effect on network segregation, but cannot confirm its segregating effect under homophily identified in previous studies^19^.  Unbiased triadic closure further reduces in-group degree inequality if links are otherwise chosen based on preferential attachment. With the exception of extreme or no homophily, in-group inequality is always higher in one of the two groups. Between-group inequality, on the other hand, disfavors the minority in homophilic settings but favors it in heterophilic ones. Preferential attachment increases between-group inequality in all cases, whereas triadic closure often reduces it. Combining in-group and between-group inequality, we find that the group accumulating more links is also the one with higher within-group inequality. Only a few nodes in the favored group receive a large share of the links, while the majority of the group is left with lower degree visibility.

Applying model inference to fifty years of empirical scientific collaboration and citation networks, we find that the model variant in which both global and triadic closure links are biased by preferential attachment and moderate gender homophily best reproduces the observed gender inequalities. Our results highlight that segregation (EI), within-group inequality (Gini), and between-group inequality (MW) capture distinct social dimensions and therefore motivate different interventions. Increasing cross-group ties may reduce segregation while creating between-group disadvantage. Reducing degree inequalities and segregation in networks following these dynamics requires simultaneously lowering both homophily and triadic closure. However, improvements to group-level inequalities can negatively impact individuals. For instance, triadic closure is linked to individual performance in scientific production and citations received^17^,  and reducing preferential attachment would naturally disfavor popular scientists. Our analysis assumes stationarity in scientific citation and collaboration behavior across the observation period. In reality, scientific norms and practices have changed, for instance, by the increasing adoption of impact quantification ^51,52^ or collaboration in teams^53^.  Subsequent works could extend the model capabilities to incorporate evolving mechanisms to better capture the evolution of scientific work practices.

By considering the presence and absence of a collection of link formation mechanisms, we disentangle potential causal drivers of each mechanism on the observed network structure. However, our model is not exhaustive. For example, we fix an identical homophily value for both groups. In reality, one group might be more homophilic than another. Empirical evidence suggests that homophilic tendencies depend on group size and can vary between groups^54^.  Homophilic preference could further differ between global and triadic closure target selection, modeling differences in how people prefer linking to others based on the context in which they meet them. This could be tied to empirical findings that suggest that homophilic tendencies decrease with network proximity^31^.  Tunable and asymmetric preferential attachment could bring the simulated degree inequality closer to that of empirical networks. Lastly, our network model is only a simplification of reality. Our model is a growth model, meaning that we only consider the formation of new links and do not alter established links. In many empirical social networks, individuals may decide to break ties with others or exit the network altogether. While improving the model’s realism is valuable, adding more parameter variations and asymmetries would also increase its complexity and thus complicate interpretation.

Mechanistic models like PATCH have the potential to explain the emergence of network structure^14^,  inequalities ^21,24^ and the effects of interventions^28^.  If policy making aims to reduce network-based inequalities, it is crucial to understand the interplay of mechanisms that cause them. In our model, triadic closure can either amplify or reduce inequality, depending on preferential attachment and homophily. The implementation of interventions, such as recommendation algorithms boosting triadic closure or preferential attachment by suggesting friends of friends, or popular people, should therefore carefully consider the present context to balance the mechanisms and desired outcomes. For example, in the presence of preferential attachment and homophily, as in academic collaboration or citation, the introduction of a triadic-closure-based recommendation algorithm could slightly reduce between-group inequality at the cost of increased within-group inequality if the algorithm was biased by homophily. In contrast, an algorithm agnostic to both popularity and homophily could potentially reduce both within-group and between-group inequality.

## Methods

### Model specification

As an extension to the Barabási-Albert model, PATCH is initialized with *m* fully connected nodes^14^. Each of the remaining $N-m$ nodes joins the network one after the other and links to *m* previously added nodes. Group assignments are drawn randomly with probability $f_{\min }$ for each node. For each link, the node can choose globally among all previously added nodes with probability $1 - \tau$, following prior triadic closure model variants. Otherwise, with triadic closure probability $\tau$, we limit the available target nodes to neighbors of neighbors^25,29,30^.  Note that, because a node initially has no connections, its first link is always global. Among the chosen target set of nodes, the selection is then based on a random (uniform) choice ($\textrm{U}$), homophily ($\textrm{H}$), or preferential attachment and homophily ($\textrm{PAH}$). The probability of forming a link between nodes *i* and available target nodes *j* is then given by

2 $$\begin{aligned} \Pi _{ij} = \frac{p_{ij}}{\sum _{n < i} p_{in}} \end{aligned}$$

where $p_{ij}$ depends on the chosen link formation mechanisms. ^55^

In the uniform case ($\textrm{U}$), we fix $p_{ij} = 1$. For preferential attachment and homophily ($\textrm{PAH}$), we follow a variant of the homophily model ^21^ and set $p_{ij} = h_{ij}k_j$, where $k_j$ is the degree of node *j* and $h_{ij}$ depends on the homophily parameter $h \in (0,1)$ and the minority/majority attribute of nodes *i* and *j*. If *i* and *j* belong to the same group, $h_{ij} = h_{ji} = h$; otherwise, $h_{ij} = 1 - h$. Values of $h > 0.5$ result in homophilic tendencies, preferring in-group links, while $h < 0.5$ leads to heterophily. Note that $h=0.5$ neutralizes the effect of homophily, corresponding to preferential attachment without homophily. This recovers the triadic closure only variants ^29,30^ and the original Barabási–Albert model ^14^ if triadic closure is also disabled ($\tau = 0$). For homophily without preferential attachment ($\textrm{H}$), we neutralize the degree effect by setting $p_{ij} = h_{ij}$. If not stated otherwise, we fix $N = 5\,000$, $f_{\min }= 0.2$, $m = 3$, simulate each model 100 times, and vary *h* and $\tau$ as control parameters. Simulating networks with PATCH scales quadratically in the number of nodes $O(N^2)$ (see Section S1 for a derivation).

Our model definition is flexible to varying link formation mechanisms $\mathrm {L_{G,T}} \in \{\textrm{U}, \textrm{H}, \textrm{PAH}\}$ when forming global $\mathrm {L_G}$ or triadic closure edges $\mathrm {L_T}$. For example, global links may follow $\textrm{PAH}$ ($\mathrm {L_G} = \textrm{PAH}$) while triadic closure links are chosen uniformly without any group or popularity preference ($\mathrm {L_T} = \textrm{U}$). To reduce the number of model combinations while still retaining the flexibility of biasing triadic closure, we restrict our analysis to models in which triadic closure links follow either the unbiased case ($\mathrm {L_T} = \textrm{U}$) or whatever mechanism is specified for global links ($\mathrm {L_T} = \mathrm {L_G}$), including the respective homophily parameterization *h*.

### Empirical networks

To account for representational and behavioral changes in time, such as the increase of the fraction of women ^12^ (see also Fig. 5a and Table S1) or decreasing gender homophily in collaboration^32^,  we consider decade-long network snapshots, starting between 1970 and 2010 for APS and APS-CIT, and between 1970 and 2000 for DBLP. In PATCH, all existing nodes are available targets for linking. Although this is realistic when citing older papers, authors may no longer be available for collaboration after retiring. In the collaboration networks (APS and DBLP), we thus only consider active authors as those who continue to publish after the end of the respective decade. We observe post-decade publications for one year in APS and eight years in DBLP; this affects only the final decade of each dataset. Each snapshot consists of the scientists for whom a gender label could be inferred from their names using an open source inference approach at a 95%-certainty threshold^56^.  While the chosen threshold of 95% is conservative, more restrictive values would quickly diminish the sample size. Similar or less restrictive levels for the same algorithm and overlapping datasets have been used by similar studies^17,57^.  The disambiguation of scientists, tracking their publications over time, is provided by rule-based solutions for the APS ^17,58^ and DBLP datasets^37^.  In the collaboration networks (APS and DBLP), the remaining authors are then linked pairwise if they co-authored a paper during the respective decade. In the citation network APS-CIT nodes are papers, linked if one paper cites another. We leave the inclusion of directionality of PATCH for future work and ignore it for this application. In Physics and Computer Science, the order of author names on a paper typically signals the authors’ contribution. Aligning with previous research, we are interested in gendered citation inequalities with regards to the first authors and thus label a paper to belong to the minority group if its first author was labelled as a female scientist^38^. 

### Likelihood-free inference

To learn the underlying link formation mechanisms that best explain the inequalities of the empirical networks, we want to estimate the posterior distribution of the model parameters *h* and $\tau$

3 $$\begin{aligned} p(h, \tau | G) = \frac{p(G | h, \tau ) p(h, \tau )}{p(G)}, \end{aligned}$$

where $p(h, \tau )$ is the prior distribution of the model parameters and $p(G | h, \tau )$ is the likelihood of the observed networks *G* given the model parameters. However, the likelihood term is intractable for our generative model. Approximate Bayesian computation (ABC) is a likelihood-free inference method that allows us to estimate the posterior distribution of the model parameters by comparing simulated and observed networks by a selection of summary statistics^59–61^.  Beyond the metrics of segregation (EI), between-group inequality (Mann–Whitney test statistic) and within-group inequalities ($\mathrm {Gini_{min}}$, and $\mathrm {Gini_{maj}}$), we inform the inference with the average local clustering coefficient (CCF)^1^.  By measuring the fraction of closed triangles, we expect the CCF to be informative about the triadic closure parameter $\tau$ which we showed to have a moderate effect on the other metrics. We use the local clustering coefficient variant to decrease the influence of the network size on the summary statistics, which we fix to $N_{\text {sim}} = 500$ nodes to balance computational costs given the quadratic scaling of $O(N^2)$ (see Section S1). Figures S1 to S6 show that the summary statistics are invariant to the network size. The global degree inequality ($\textrm{Gini}$) is not used for the inference to reduce the focus on degree-based inequalities as they are already captured by the two within-group measures. The number of links per node *m* matches the average degree of the empirical networks with a lower bound of $m \ge 2$ (see Section S3). The prior distribution is uniform for both parameters $h,\tau \sim \textrm{Uniform}(0, 1)$ and the minority fraction $f_{\min }$ is chosen directly from the empirical networks (see Fig. 5a). Using the elfi Python package^62^,  we apply seven rounds of Sequential Monte Carlo sampling with adaptive distance weighting ^63^ to draw 1000 posterior samples of the model parameters *h* and $\tau$.

We evaluate this setup using a three-step process. The first two steps apply a synthetic evaluation. We simulate PATCH networks with varying model variants and known parameters *h* and $\tau$, averaging the summary statistics over 100 simulations. In the first evaluation step, we aim to retrieve the model variant used during the simulations. Each fit yields a distribution of distances between the observed summary statistics and the statistics corresponding to the accepted posterior sample. We select the model variant with the lowest distance distribution compared to the unified distance distribution of all other variants. Our approach correctly classifies most model variants, but confuses local link formation mechanisms in the absence of triadic closure, or under extreme or neutral homophily values (see Figure S17 and its caption for a discussion).

The second step evaluates whether the inferred posterior distributions of the selected model variant center around the true parameters. We identify good agreement for all simulated variants, with increasing uncertainty for more expressive models which include $\textrm{PAH}$ in its link formation mechanisms (Figs. S18 to S21).

As a third evaluation step, we perform predictive checks to test whether the selected inferred model can reproduce the empirical network inequalities. We compute summary statistics averaged across 100 PATCH network simulations for each of the 1000 posterior sample pairs $(h, \tau )$ and compare them to the empirical statistics. Although PATCH can reproduce empirical segregation, between-group inequality, and clustering surprisingly well (Figures S22, S25 and S26), the within-group degree inequalities are not well captured (Figures S23 and S24). Because the strength of preferential attachment is fixed in PATCH, it cannot match the strong degree inequality in the empirical networks. However, the model reproduces the relative ratio of degree inequality between the two groups, even if the measure was not used for the inference (Fig. S27). Given PATCH’s simplicity, we consider this a reasonable result which also informs us about model limitations and the need for further model extensions.

## Supplementary Information

Supplementary Information.

## Data Availability

The APS and APS-CIT datasets^36^ are available by request from https://journals.aps.org/datasets/aps-cit. The DBLP dataset^37,64^ is freely available at https://dblp.org/. For this analysis, a pre-processed network version of DBLP is used, which is available through the konect network repository^65^ at http://konect.cc/networks/dblp_coauthor/. The code to reproduce the results of this paper is available at https://github.com/mannbach/patch. The repository is linked to a Zenodo archive at 10.5281/zenodo.17160884, which contains the simulated networks and aggregated statistics. The PATCH model is further implemented in version 2.0.0a2 of the netin Python package, which is available at https://pypi.org/project/netin/2.0.0a2/.

## Code availability

The code to reproduce the results of this paper is available at https://github.com/mannbach/patch. The repository is linked to a Zenodo archive at 10.5281/zenodo.17160884, which contains the simulated networks and aggregated statistics. The PATCH model is further implemented in version 2.0.0a2 of the netin Python package, which is available at https://pypi.org/project/netin/2.0.0a2/.
