# Supplementary material for: Network inequality through preferential attachment, triadic closure, and homophily
Source: Sci Rep. 2026 Mar 13;16:13461. doi: 10.1038/s41598-026-42911-3 (PMC13111714; doi:10.1038/s41598-026-42911-3)
Supplement: Supplementary file 1 — Supplementary Information. [file 41598_2026_42911_MOESM1_ESM.pdf]

Supplementary Information for “Network Inequality through Preferential Attachment, Triadic Closure, and Homophily”

Jan Bachmann[1,2,3,*], Samuel Martin-Gutierrez[3,4,†], Lisette Espín-Noboa[1,2,†], Nicola Cinardi[5,6], and Fariba Karimi[3,1,*]

[1]Complexity Science Hub, 1030 Vienna, Austria
[2]Department of Network and Data Science, Central European University, 1100 Vienna, Austria
[3]Graz University of Technology, 8010 Graz, Austria
[4]Grupo de Sistemas Complejos, ETS de Arquitectura de Madrid, Universidad Politécnica de Madrid, 28040 Madrid, Spain
[5]Faculty of Engineering, Free University of Bozen-Bolzano, 39100 Bolzano, Italy
[6]Center for Computational and Stochastic Mathematics, Instituto Superior Tecnico, Universidade de Lisboa, 1049-001 Lisbon, Portugal
[*]*Corresponding authors email: bachmann@csh.ac.at, karimi@tugraz.at*
[†]*These authors contributed equally to this work.*

Supplementary information

S1 Computational complexity

We estimate the computational cost of simulating a PATCH network with N nodes where each new node forms m links, creating $O(Nm)$ edges in total. Each link is either global (with probability $1-\tau$) or formed via triadic closure (with probability τ).
Global links. When forming a global link, the new node chooses among $O(N)$ existing nodes. Evaluating and normalizing the weights p_{ij} requires iterating over all candidates, which takes $O(N)$ time per global link. With $O(Nm)$ global links in total, this yields $O(N^2m)$.
Triadic closure links. When forming a triadic closure link, the candidate set is restricted to friends-of-friends. If the new node i has current degree $k_i \leq m$, enumerating friends-of-friends requires iterating over neighbors-of-neighbors, which costs

$$O\left(\sum_{u\in\mathcal{N}(i)} k_u\right), \tag{1}$$

where $\mathcal{N}(i)$ denotes the neighbors of i and k_u is the degree of neighbor u. Using $\sum_{u\in\mathcal{N}(i)} k_u \approx k_i\langle k\rangle$ and the average degree $\langle k\rangle = 2L/N \approx 2m$, the expected cost per triadic closure link is $O(m^2)$. With $O(Nm)$ triadic closure links in total, this yields $O(Nm^3)$.
Taken together, the total time complexity is

$$O(N^2m + Nm^3), \tag{2}$$

which reduces to $O(N^2)$ for fixed $m \ll N$.

S2 Theoretical EI-index

The theoretical EI-index in the absence of homophily biases in link formation can be derived from the expected number of cross-group and in-group links

$$\mathbb{E}[\mathrm{EI}] = \mathbb{E}\left[\frac{E-I}{E+I}\right] = \frac{\mathbb{E}[E]-\mathbb{E}[I]}{L}, \tag{3}$$

as the total number of links $L = E + I$ is deterministic.

The expected number of cross-group links E can be computed by considering that each of the L links has a probability of $2f_{\min}(1-f_{\min})$ to connect nodes of different groups

$$\mathbb{E}[E] = 2f_{\min}(1-f_{\min})L. \tag{4}$$

The expected number of in-group links I is given by

$$\mathbb{E}[I] = [f_{\text{min}}^2 + (1 - f_{\text{min}})^2]L \tag{5}$$

Combining these two expressions yields the theoretical EI-index

$$\mathbb{E}[\text{EI}] = \frac{2f_{\text{min}}(1 - f_{\text{min}})L - (1 - 2f_{\text{min}}(1 - f_{\text{min}}))L}{L} \tag{6}$$

$$= 4f_{\text{min}}(1 - f_{\text{min}}) - 1 = -(2f_{\text{min}} - 1)^2 \leq 0. \tag{7}$$

This last expression demonstrates that $\mathbb{E}[\text{EI}] \leq 0$ independently of group sizes if link formation is random and unbiased, and it is only 0 when group sizes are equal ($f_{\text{min}} = 0.5$).

S3 Inferring m

We infer the number of links per node m used in the inference simulations by matching the average degree between the observed empirical and the simulated networks $\langle k_{\text{emp}} \rangle \approx \langle k_{\text{sim}} \rangle$. The average simulation degree

$$\langle k_{\text{sim}} \rangle = \frac{2L_{\text{sim}}}{N_{\text{sim}}} \tag{8}$$

depends on the total number of nodes N_{sim} and links L_{sim}. To balance computational costs, we fix the number of nodes to $N_{\text{sim}} = 500$. The number of links L_{sim} in PATCH depends on the number of links per node m and the number of nodes N_{sim} as

$$L_{\text{sim}} = \underbrace{\frac{m(m-1)}{2}}_{\text{initial clique}} + \underbrace{(N_{\text{sim}} - m)m}_{\text{added nodes}} \tag{9}$$

$$= mN_{\text{sim}} - \frac{m(m+1)}{2}. \tag{10}$$

We infer m by equalizing the average degree of the empirical networks $\langle k_{\text{emp}} \rangle$ with the average degree of the simulated networks $\langle k_{\text{sim}} \rangle$

$$\langle k_{\text{emp}} \rangle = \langle k_{\text{sim}} \rangle \tag{11}$$

$$= \frac{2}{N_{\text{sim}}} L_{\text{sim}}. \tag{12}$$

Substituting the expression for L_{sim} and rearranging the terms yields

$$\frac{2}{N_{\text{sim}}}(mN_{\text{sim}} - \frac{m(m+1)}{2}) - \langle k_{\text{emp}} \rangle = 0 \tag{13}$$

$$2m - \frac{m(m+1)}{N_{\text{sim}}} - \langle k_{\text{emp}} \rangle = 0 \tag{14}$$

Multiplying by N_{sim} and additional rearranging gives the quadratic equation

$$m^2 - m(2N_{\text{sim}} - 1) + \langle k_{\text{emp}} \rangle N_{\text{sim}} = 0 \tag{15}$$

which we solve for m using the quadratic formula

$$m_{+,-} = \frac{1}{2}\left(2N_{\text{sim}} - 1 \pm \sqrt{(2N_{\text{sim}} - 1)^2 - 4\langle k_{\text{emp}} \rangle N_{\text{sim}}}\right). \tag{16}$$

Because $m \leq N_{\text{sim}}$ is a strict upper bound for m, we only consider the subtraction solution. As triadic closure links are only formed between neighbors of neighbors, we finally require $m \geq 2$

$$m = \max\left(2,\ \frac{1}{2}\left[2N_{\text{sim}} - 1 - \sqrt{(2N_{\text{sim}} - 1)^2 - 4\langle k_{\text{emp}} \rangle N_{\text{sim}}}\right]\right), \tag{17}$$

and round the solution to the nearest integer.

Supplementary tables

S1 Empirical network summary statistics

Source	Decade	N	L	$\langle k \rangle$	m	$f_{\min}$	EI	$\text{Gini}_{\min}$	Gini_{maj}	MW	CCF
APS	1970	5,774	5,656	1.96	2	0.04	-0.88	0.53	0.59	0.47	0.20
	1980	11,168	16,156	2.89	2	0.06	-0.83	0.49	0.56	0.46	0.28
	1990	21,547	45,009	4.18	2	0.08	-0.77	0.50	0.55	0.46	0.35
	2000	32,374	95,455	5.90	3	0.10	-0.69	0.52	0.54	0.47	0.40
	2010	19,272	56,644	5.88	3	0.11	-0.68	0.53	0.54	0.46	0.37
APS-CIT	1970	44,461	200,067	9.00	5	0.03	-0.92	0.49	0.47	0.46	0.27
	1980	75,200	363,123	9.66	5	0.04	-0.89	0.47	0.47	0.46	0.27
	1990	133,185	746,643	11.21	6	0.05	-0.85	0.45	0.48	0.47	0.27
	2000	222,974	1,556,833	13.96	7	0.07	-0.79	0.44	0.48	0.49	0.26
	2010	321,935	2,645,852	16.44	8	0.08	-0.74	0.45	0.49	0.49	0.24
DBLP	1970	4,991	5,705	2.29	2	0.05	-0.83	0.38	0.41	0.46	0.27
	1980	21,805	32,110	2.95	2	0.09	-0.73	0.41	0.45	0.48	0.35
	1990	112,356	185,876	3.31	2	0.16	-0.68	0.48	0.52	0.43	0.31
	2000	208,854	642,096	6.15	3	0.16	-0.56	0.53	0.54	0.46	0.45

Table S1. Empirical network summary statistics. N: number of nodes; L: number of links; $\langle k \rangle$: average degree; m: inferred number of links per new node; $f_{\min}$: minority fraction; EI: EI-index; $\text{Gini}_{\min}$: Gini coefficient of the minority group; Gini_{maj}: Gini coefficient of the majority group; MW: Mann-Whitney test statistic; CCF: average clustering coefficient. APS: American Physical Society collaboration network; APS-CIT: American Physical Society citation network; DBLP: Computer Science collaboration network.

Supplementary figures

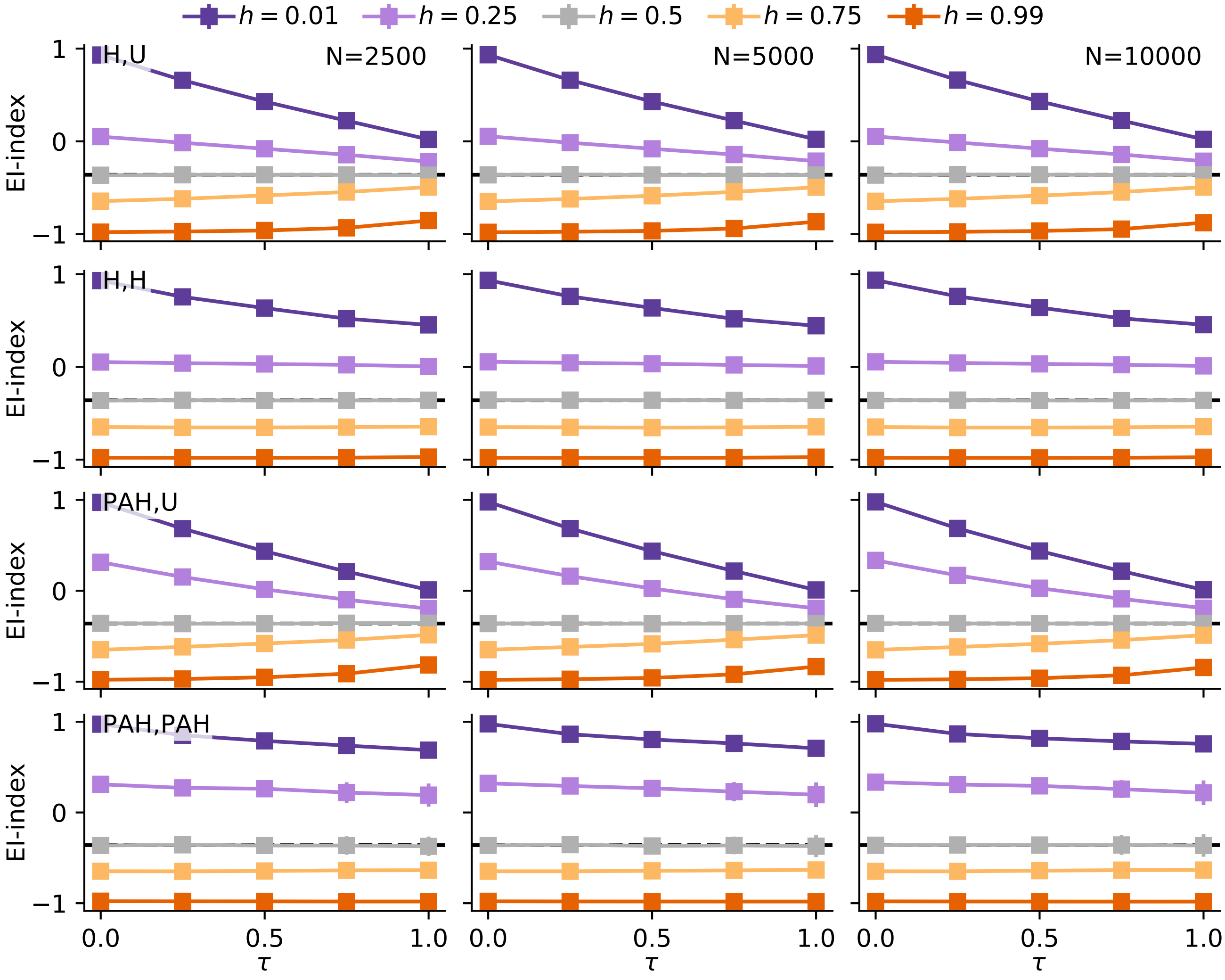

Figure S1. Sensitivity of the EI**-index to network size** N. Columns vary the number of nodes N; rows indicate model variants. Homophily h is shown by color and triadic closure τ is on the x-axis. Simulation results for the EI-index are robust to changes in N.

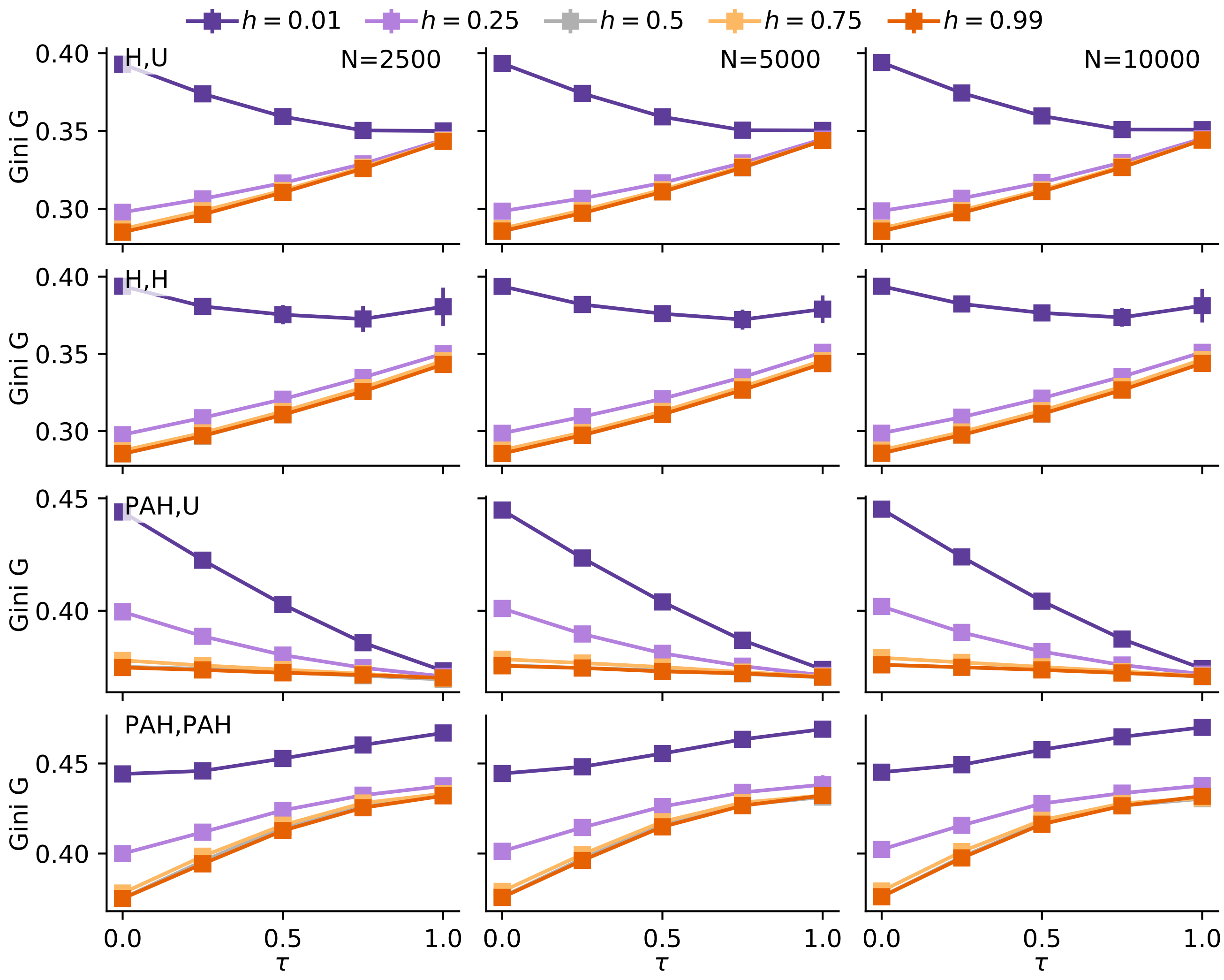

Figure S2. Sensitivity of the Gini **coefficient to network size** N. Columns vary the number of nodes N; rows indicate model variants. Homophily h is shown by color and triadic closure τ is on the x-axis. Simulation results for the Gini coefficient are robust to changes in N.

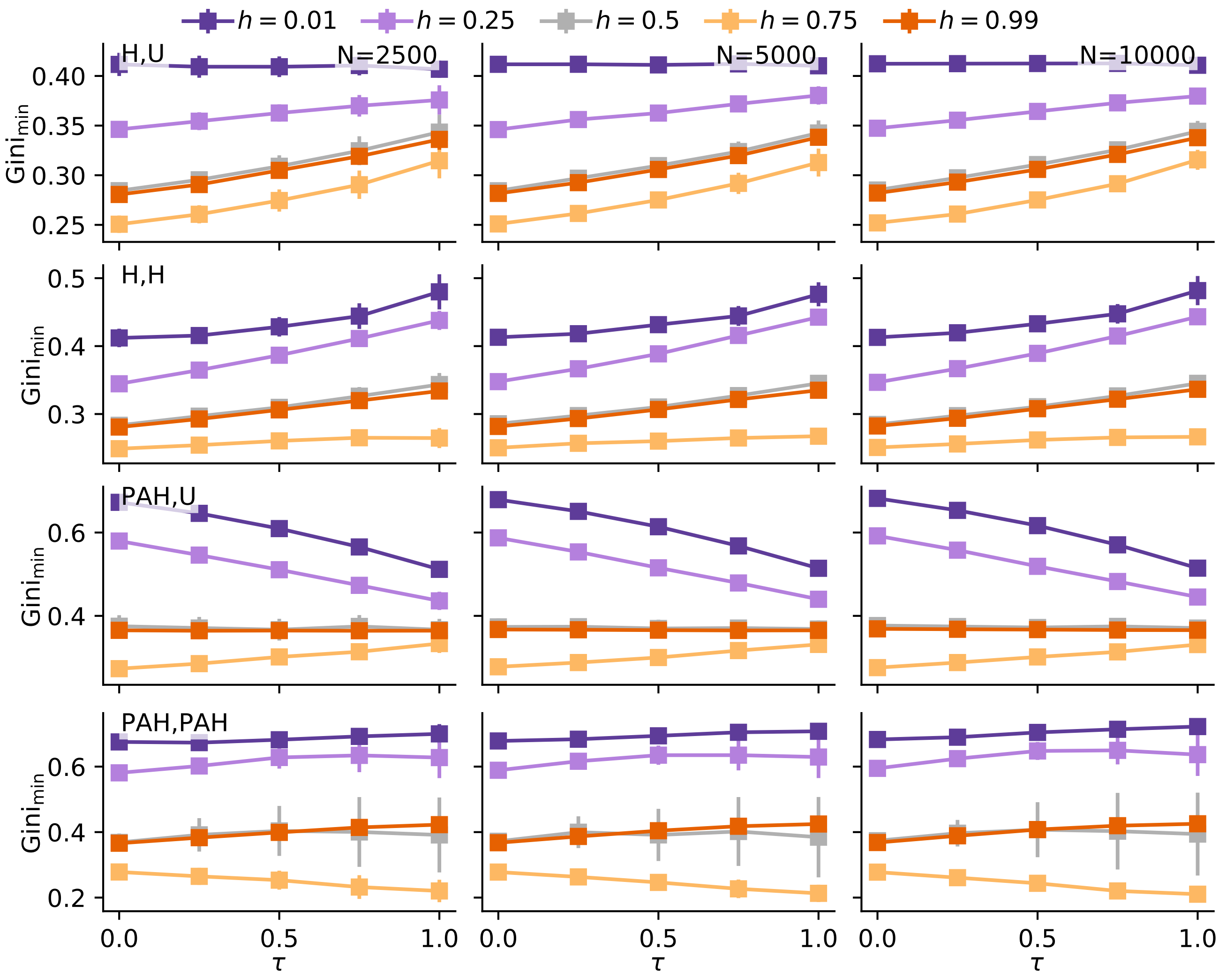

Figure S3. Sensitivity of the $\mathrm{Gini}_{\mathrm{min}}$ **coefficient to network size** N. Columns vary the number of nodes N; rows indicate model variants. Homophily h is shown by color and triadic closure τ is on the x-axis. Simulation results for the $\mathrm{Gini}_{\mathrm{min}}$ coefficient are robust to changes in N.

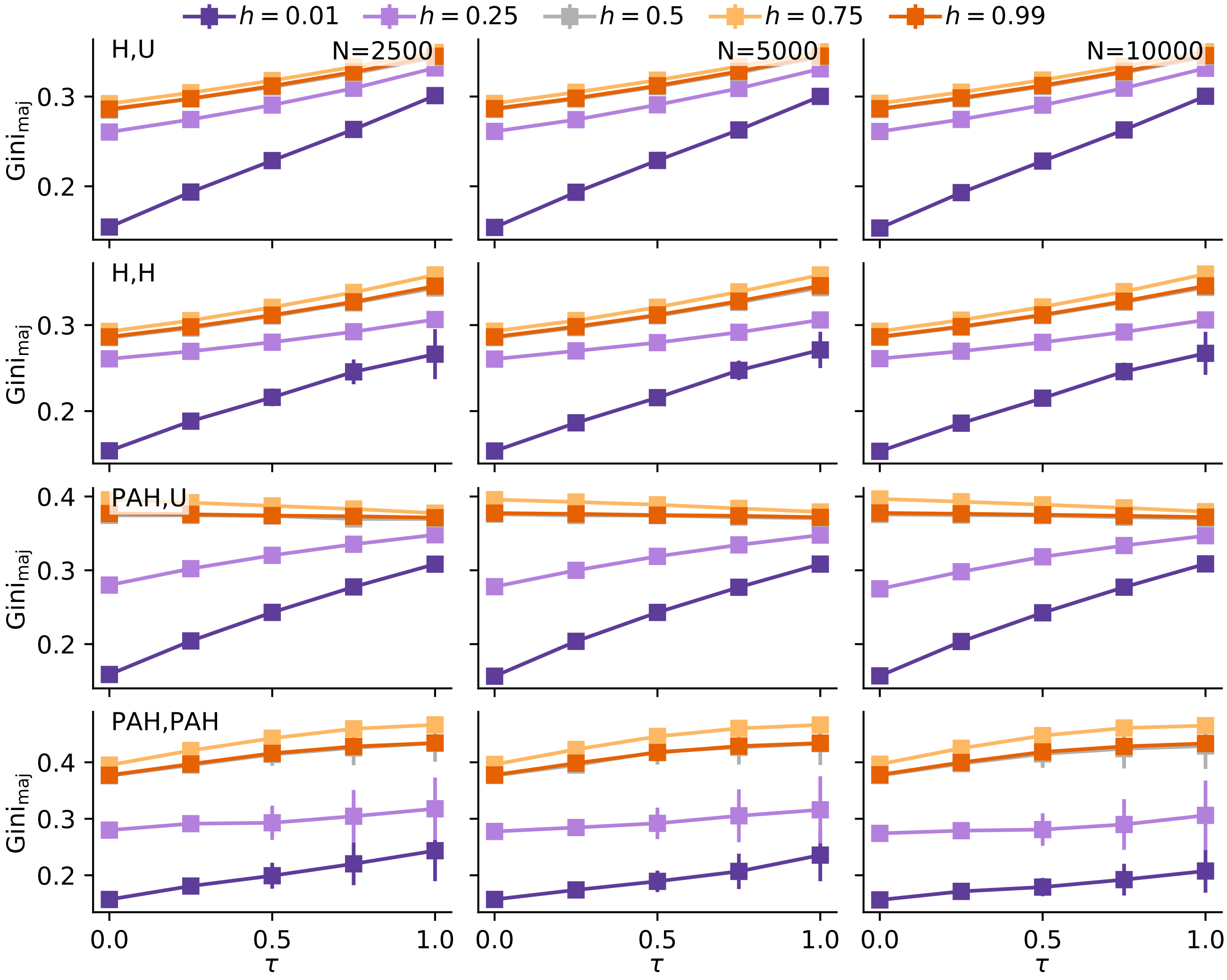

Figure S4. Sensitivity of the Gini$_{\text{maj}}$ **coefficient to network size** N. Columns vary the number of nodes N; rows indicate model variants. Homophily h is shown by color and triadic closure τ is on the x-axis. Simulation results for the Gini$_{\text{maj}}$ coefficient are robust to changes in N.

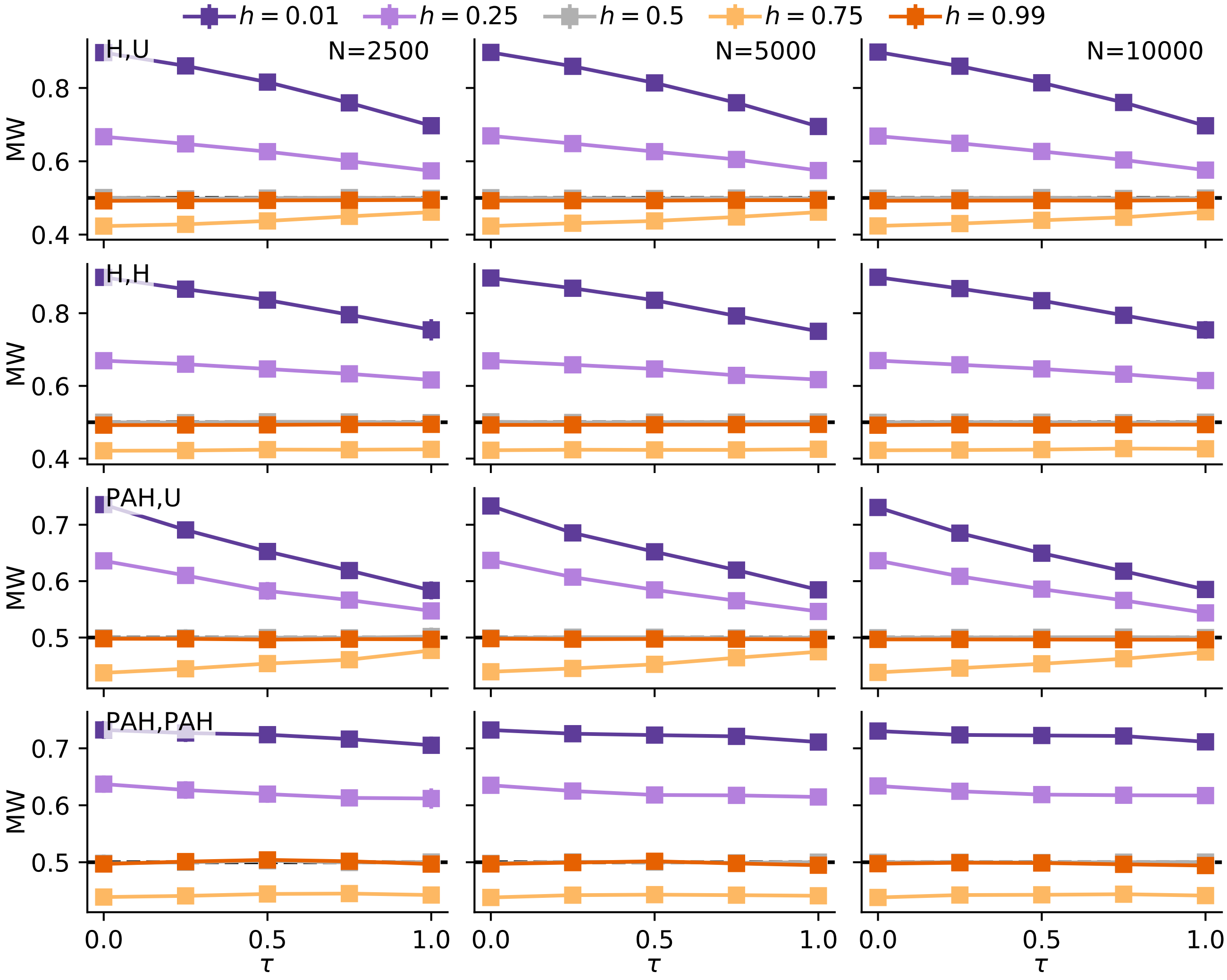

Figure S5. **Sensitivity of the Mann-Whitney test statistic to network size** N. Columns vary the number of nodes N; rows indicate model variants. Homophily h is shown by color and triadic closure τ is on the x-axis. Simulation results for the Mann-Whitney test statistic are robust to changes in N.

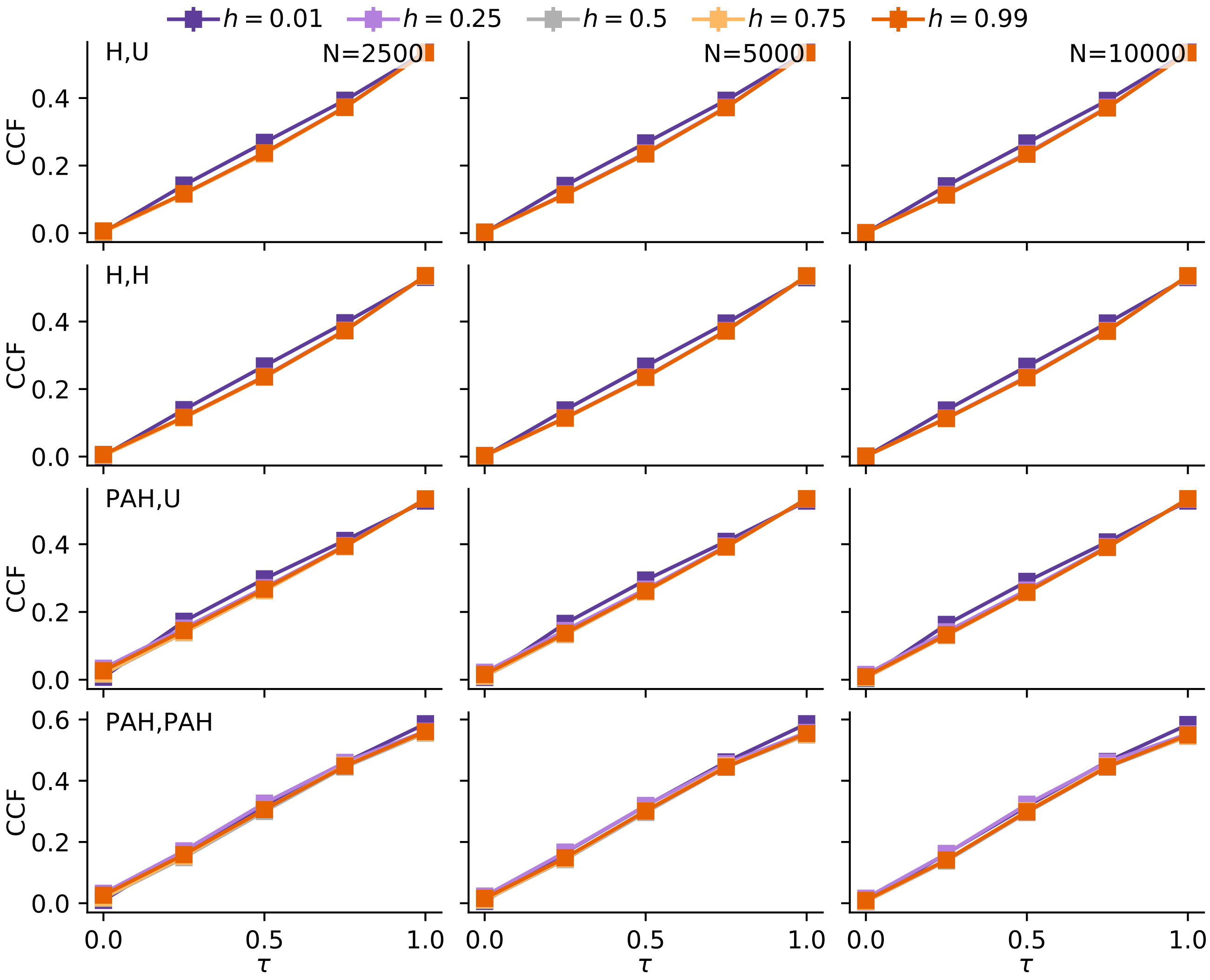

Figure S6. **Sensitivity of the average clustering coefficient (CCF) to network size** N. Columns vary the number of nodes N; rows indicate model variants. Homophily h is shown by color and triadic closure τ is on the x-axis. Simulation results for the clustering coefficient (CCF) are robust to changes in N.

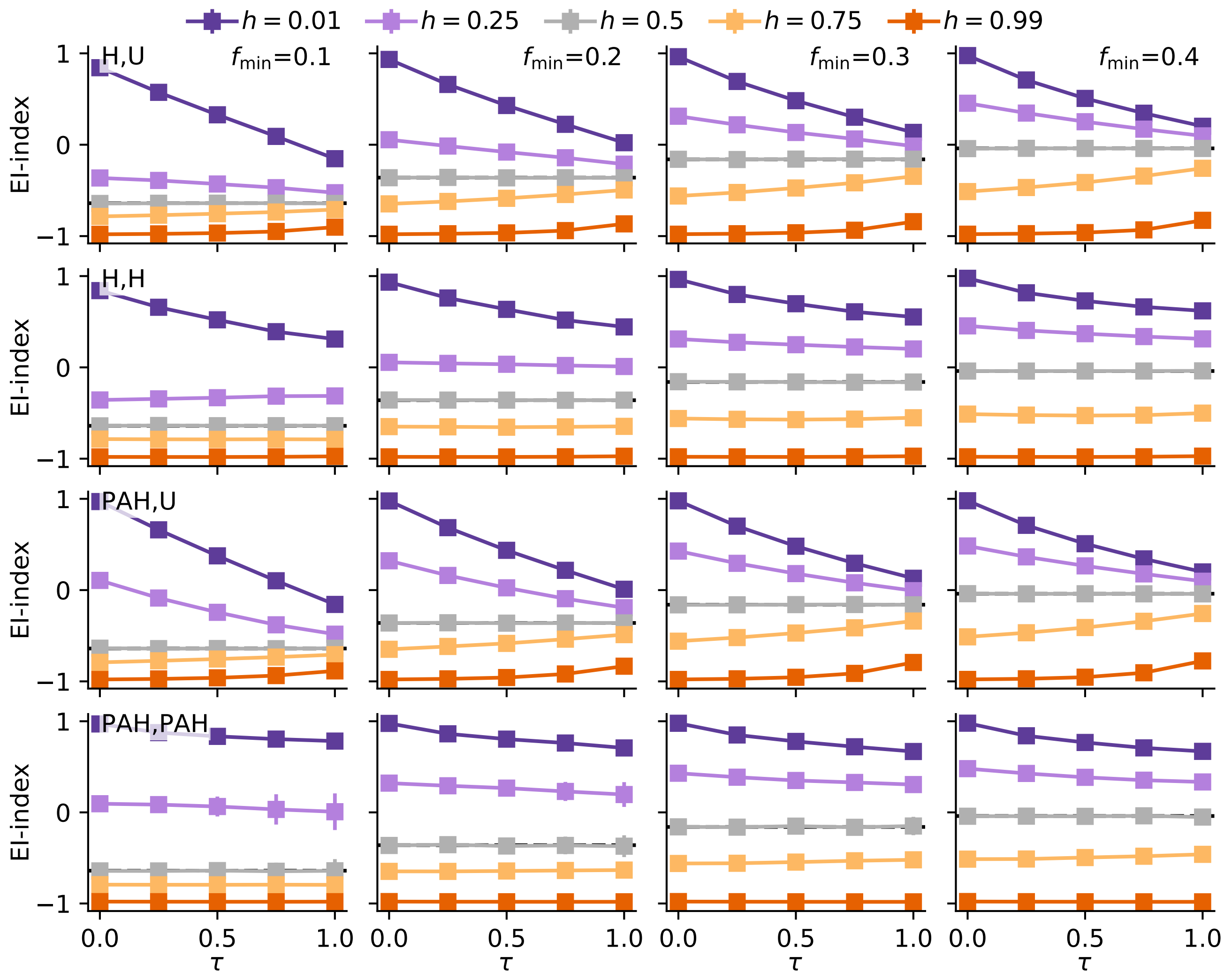

Figure S7. **Sensitivity of the EI-index to minority fraction** f_{min}. Columns vary the minority fraction f_{min}; rows indicate model variants. Homophily h is shown by color and triadic closure τ is on the x-axis. Simulation results for the EI-index are qualitatively robust to changes in f_{min} up to an offset of the neutral baseline.

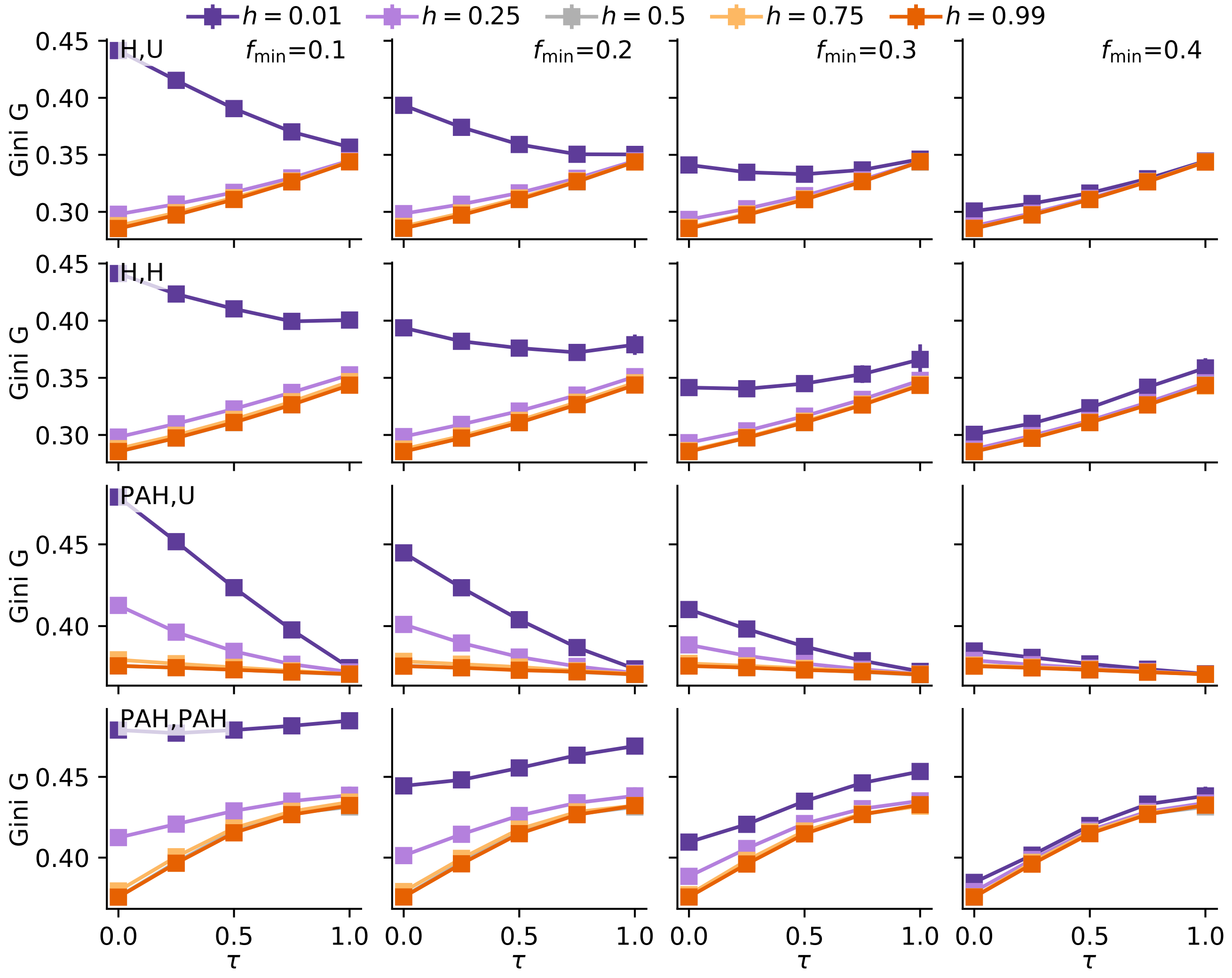

Figure S8. **Sensitivity of the** Gini **coefficient to minority fraction** f_{min}. Columns vary the minority fraction f_{min}; rows indicate model variants. Homophily h is shown by color and triadic closure τ is on the x-axis. Simulation results for the Gini coefficient are robust to changes in f_{min} with reduced effects of h and sometimes τ with increasing f_{min}.

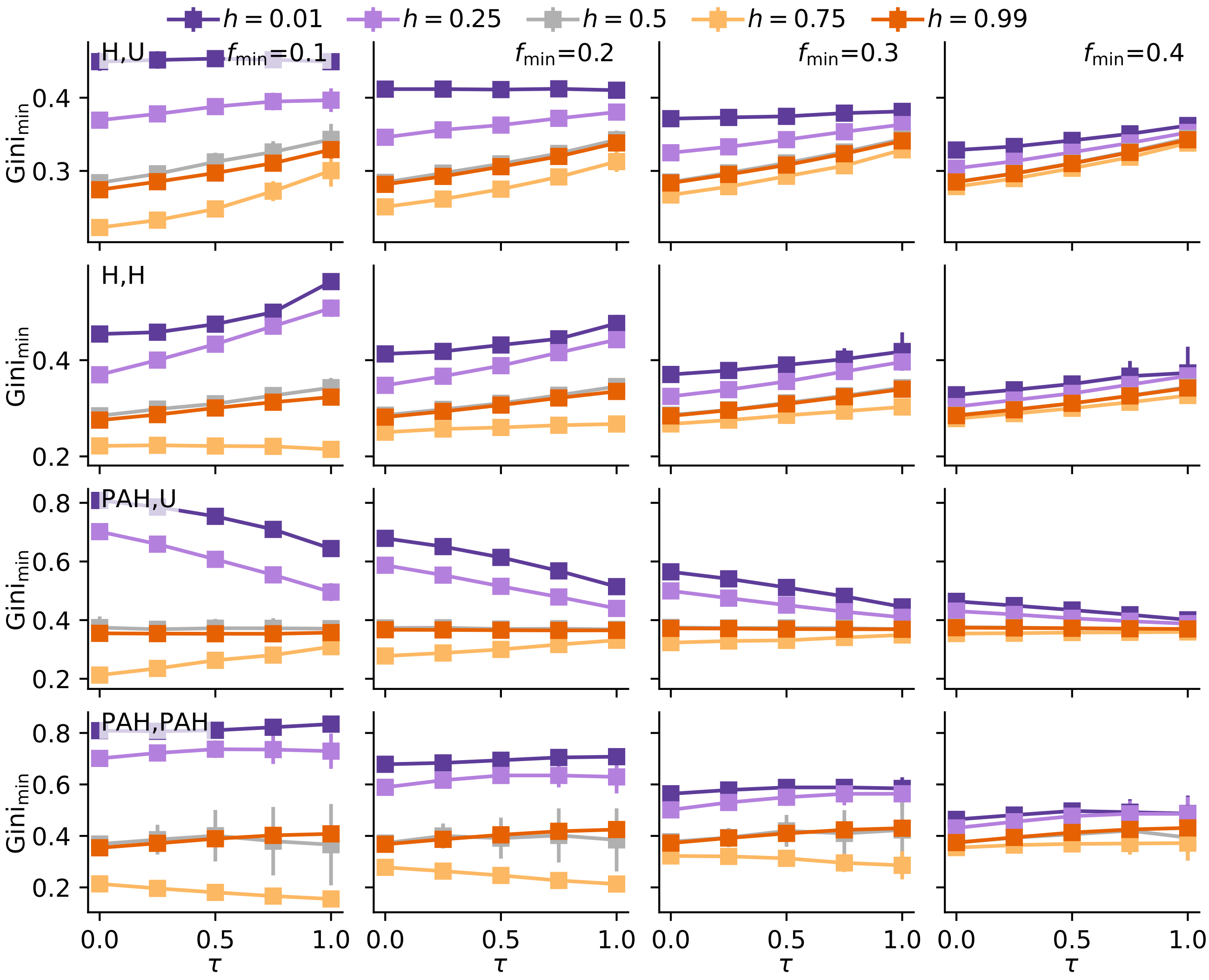

Figure S9. Sensitivity of the $\mathrm{Gini}_{\mathrm{min}}$ **coefficient to minority fraction** f_{min}. Columns vary the minority fraction f_{min}; rows indicate model variants. Homophily h is shown by color and triadic closure τ is on the x-axis. Simulation results for the $\mathrm{Gini}_{\mathrm{min}}$ coefficient are robust to changes in f_{min} with reduced effects of h and sometimes τ with increasing f_{min}.

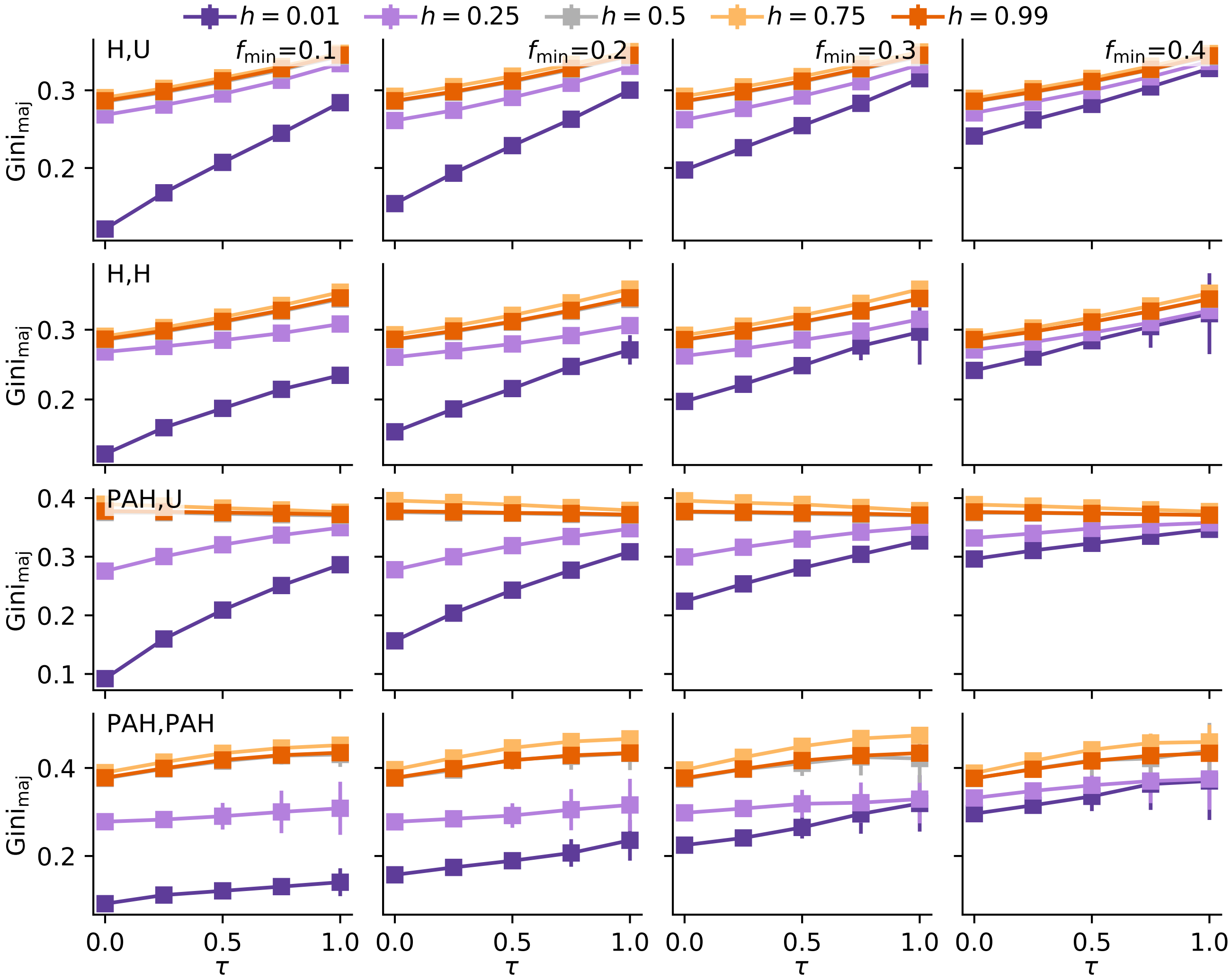

Figure S10. **Sensitivity of the** Gini_{maj} **coefficient to minority fraction** f_{min}. Columns vary the minority fraction f_{min}; rows indicate model variants. Homophily h is shown by color and triadic closure τ is on the x-axis. Simulation results for the Gini_{maj} coefficient are robust to changes in f_{min} with reduced effects of heterophilic h with increasing f_{min}.

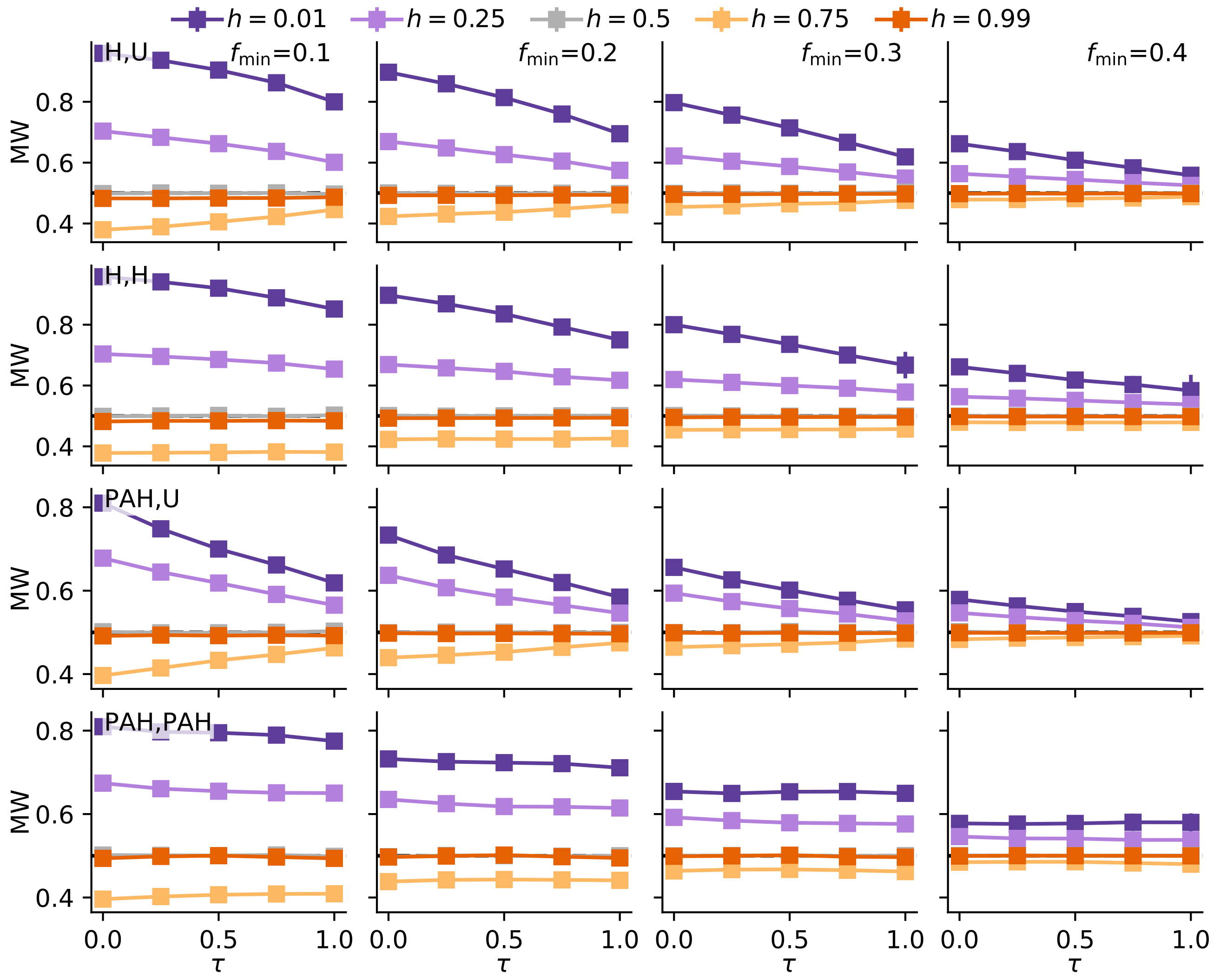

Figure S11. **Sensitivity of the Mann-Whitney test statistic to minority fraction** f_{min}. Columns vary the minority fraction f_{min}; rows indicate model variants. Homophily h is shown by color and triadic closure τ is on the x-axis. Simulation results for the Mann-Whitney test statistic are robust to changes in f_{min} with reduced effects of h with increasing f_{min}.

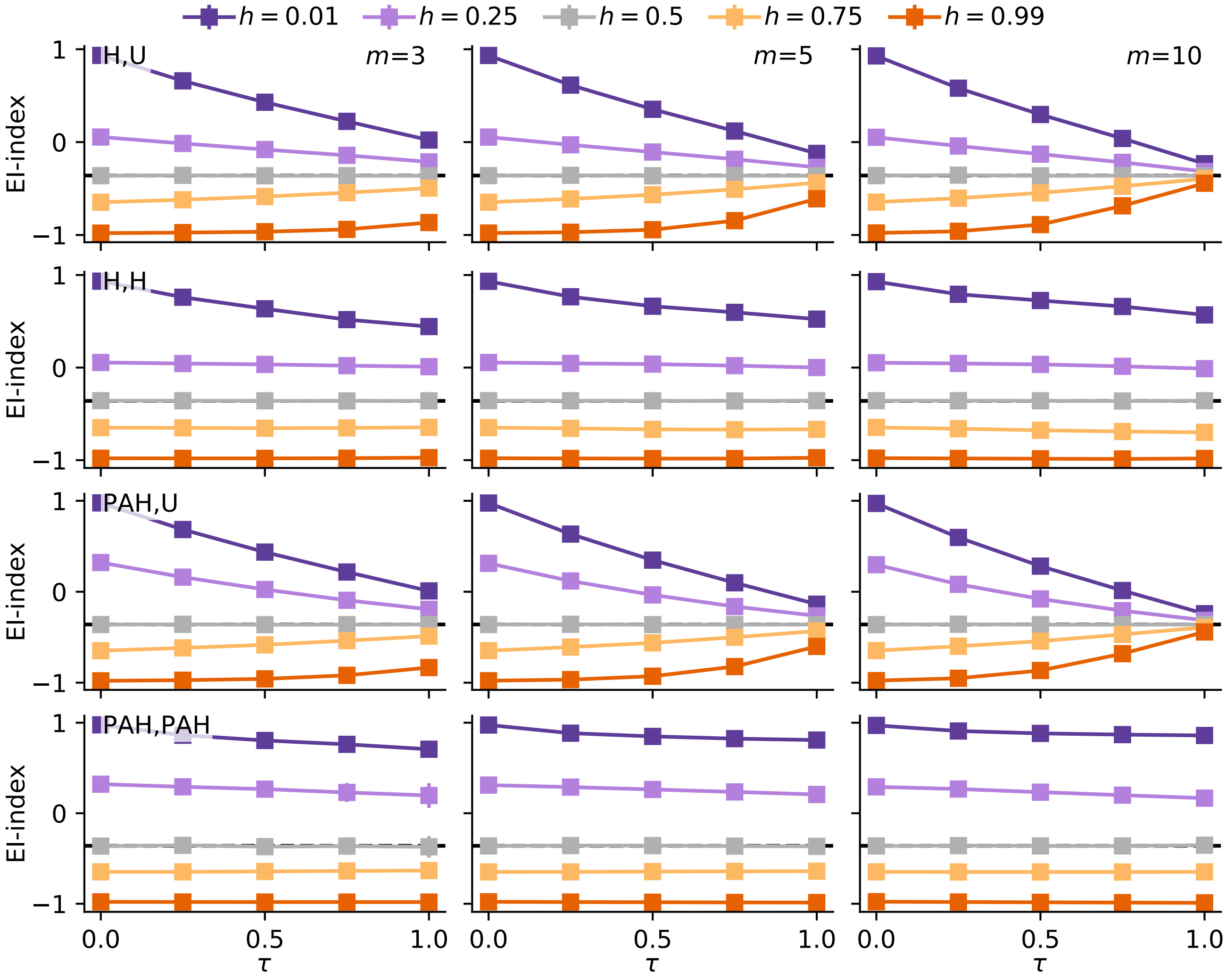

Figure S12. **Sensitivity of the EI-index to the number of new links per node m.** Columns vary the number of new links per node m; rows indicate model variants. Homophily h is shown by color and triadic closure τ is on the x-axis. Simulation results for the EI-index are robust to changes in m with the mitigation effect of unbiased triadic closure (τ) becoming stronger for larger m.

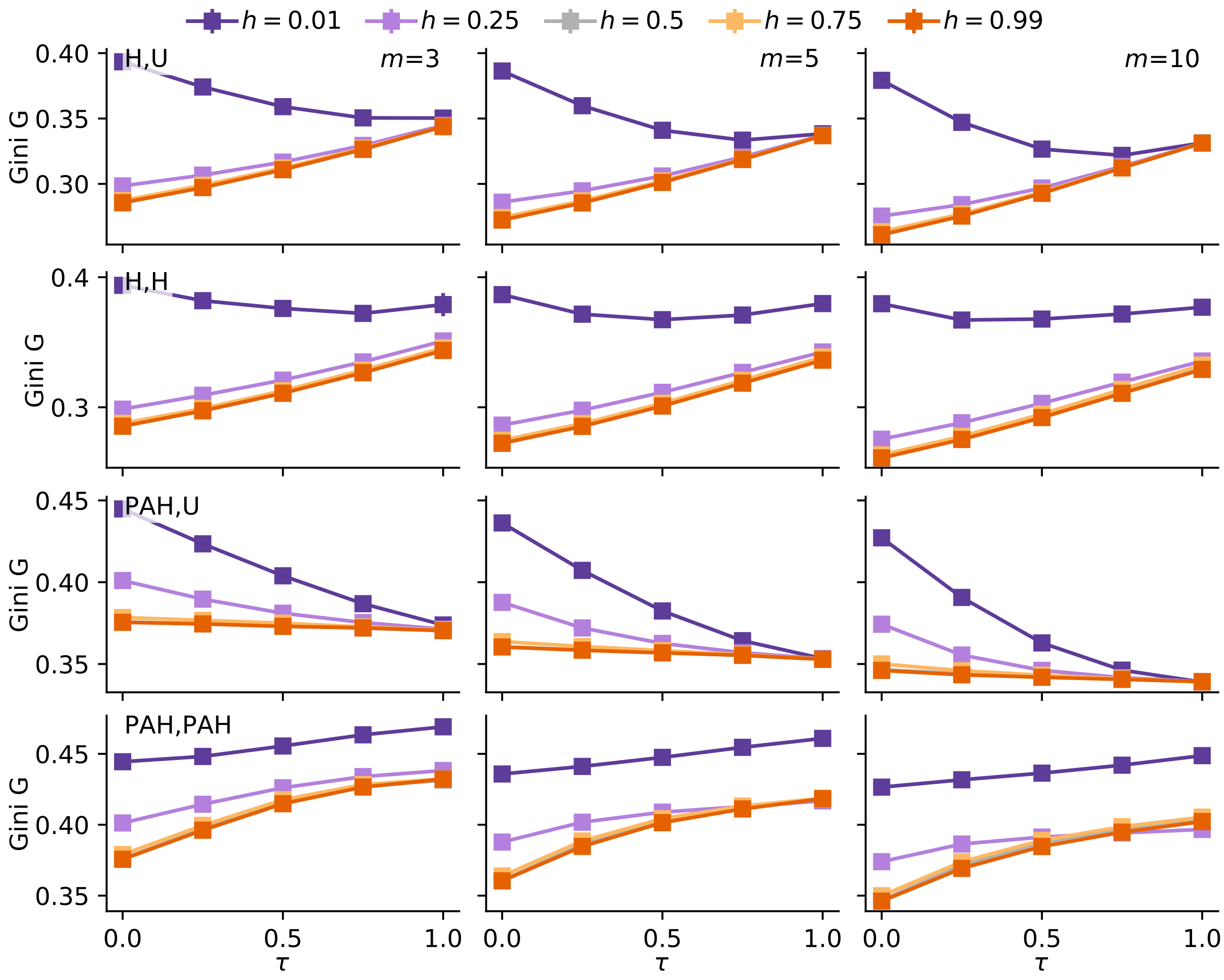

Figure S13. **Sensitivity of the** Gini **coefficient to the number of new links per node** *m*. Columns vary the number of new links per node *m*; rows indicate model variants. Homophily *h* is shown by color and triadic closure τ is on the x-axis. Simulation results for the Gini coefficient are robust to changes in *m* with the offset decreasing for larger *m*.

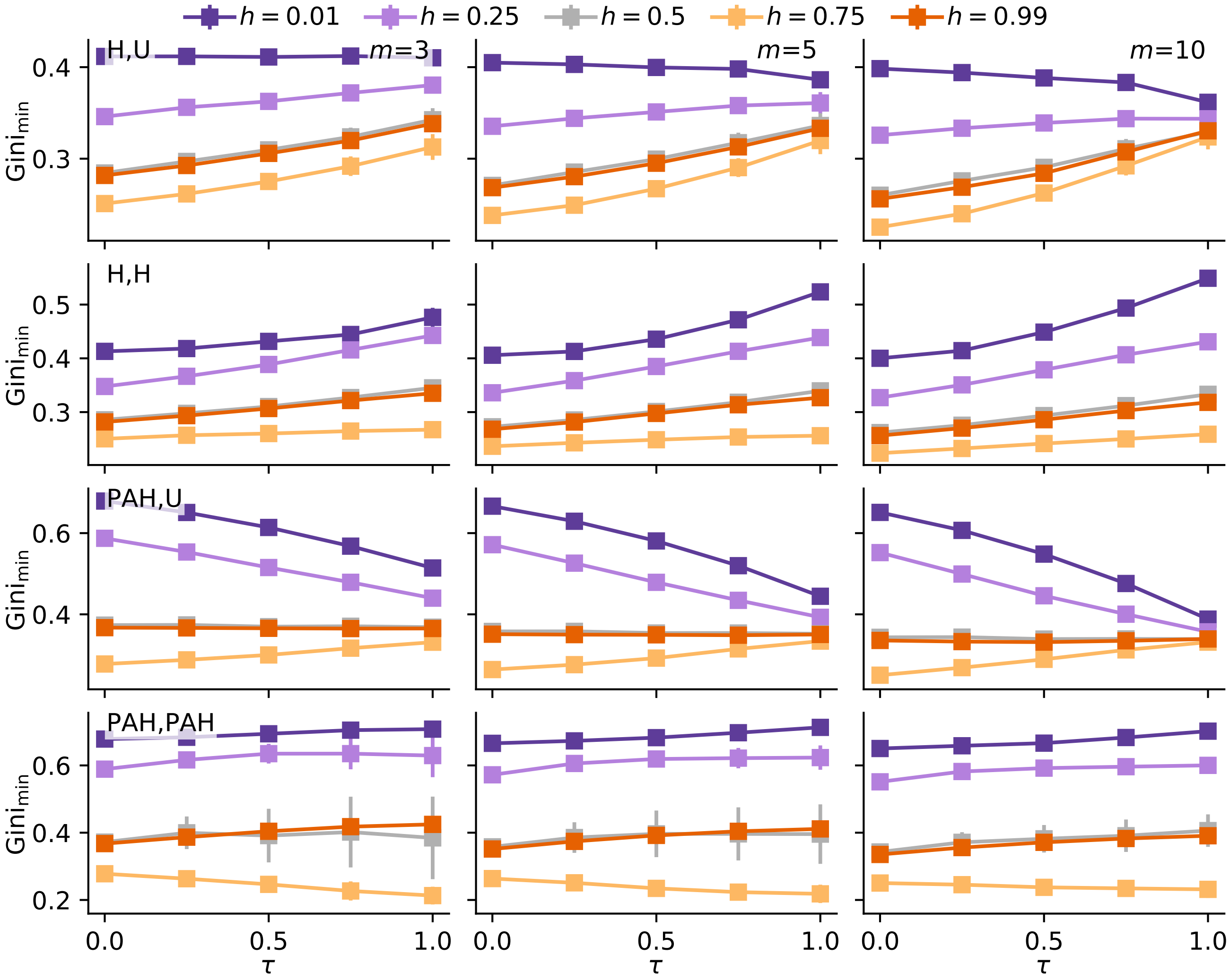

Figure S14. **Sensitivity of the** $\mathrm{Gini}_{\mathrm{min}}$ **coefficient to the number of new links per node** m. Columns vary the number of new links per node m; rows indicate model variants. Homophily h is shown by color and triadic closure τ is on the x-axis. Simulation results for the $\mathrm{Gini}_{\mathrm{min}}$ coefficient are robust to changes in m with a stronger effect of τ for unbiased triadic closure and larger m.

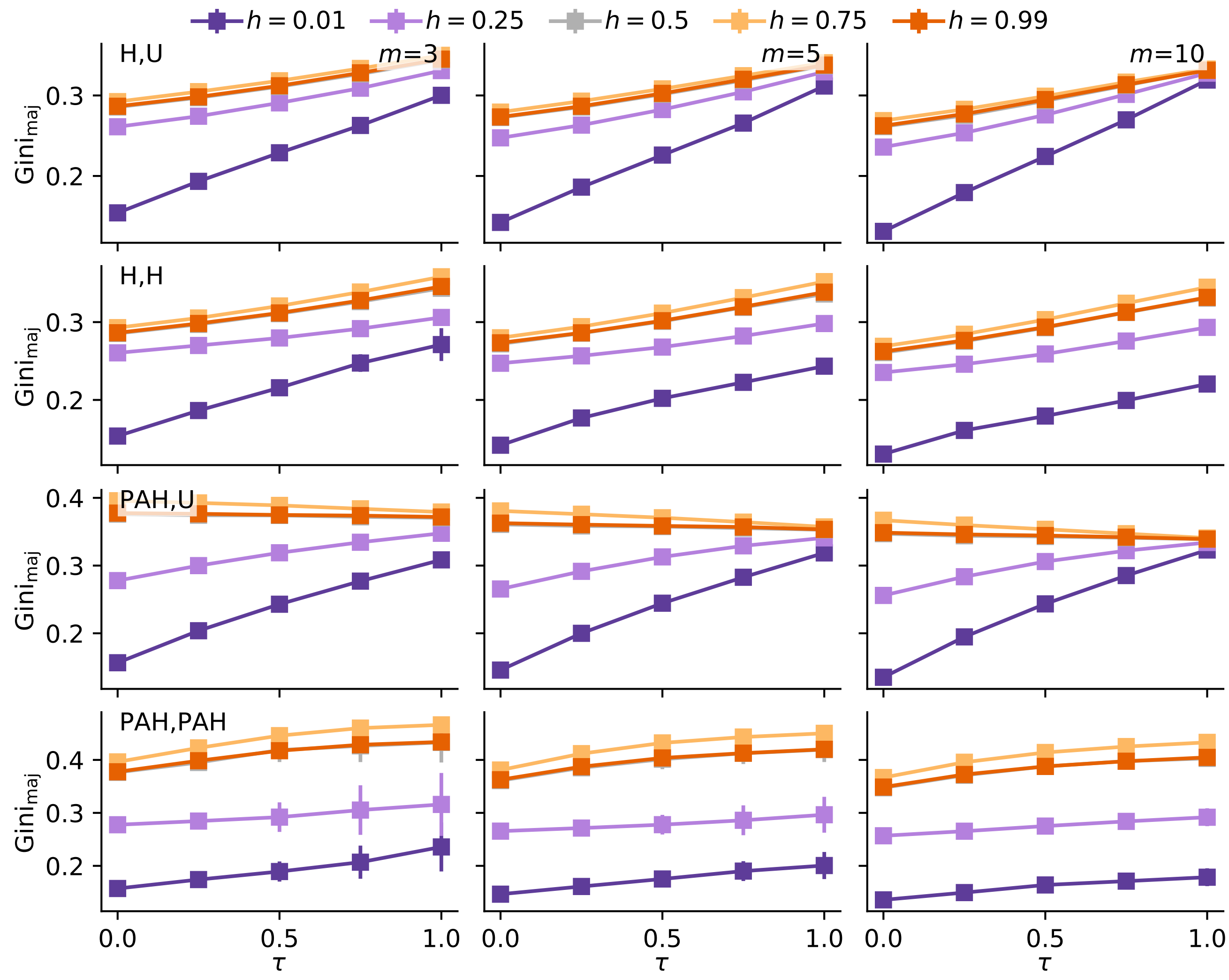

Figure S15. **Sensitivity of the** Gini$_{maj}$ **coefficient to the number of new links per node** m. Columns vary the number of new links per node m; rows indicate model variants. Homophily h is shown by color and triadic closure τ is on the x-axis. Simulation results for the Gini$_{maj}$ coefficient are robust to changes in m with a slightly lower offset for larger m.

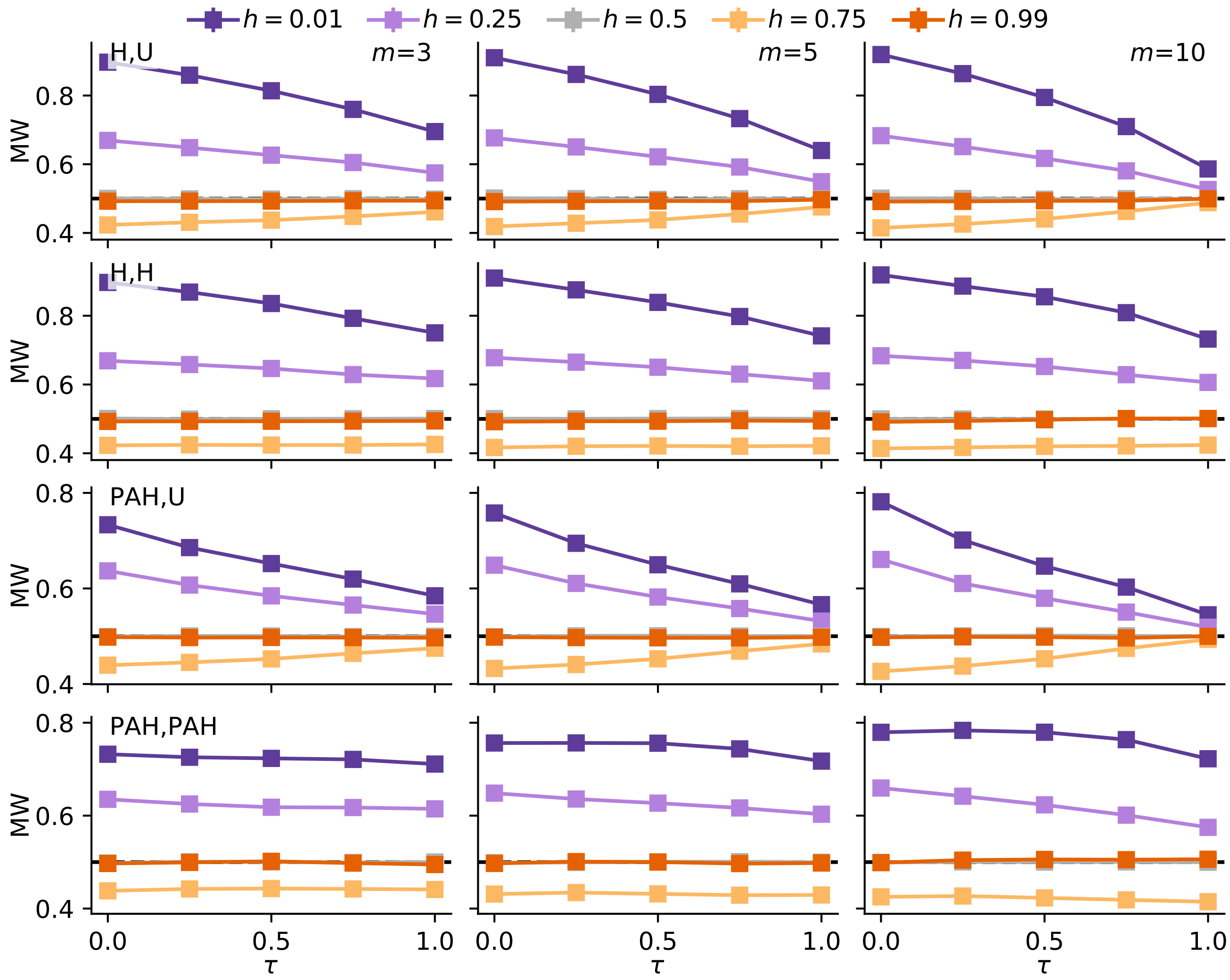

Figure S16. **Sensitivity of the Mann-Whitney test statistic to the number of new links per node** m. Columns vary the number of new links per node m; rows indicate model variants. Homophily h is shown by color and triadic closure τ is on the x-axis. Simulation results for the Mann-Whitney test statistic are robust to changes in m with a stronger effect of τ for unbiased triadic closure and larger m.

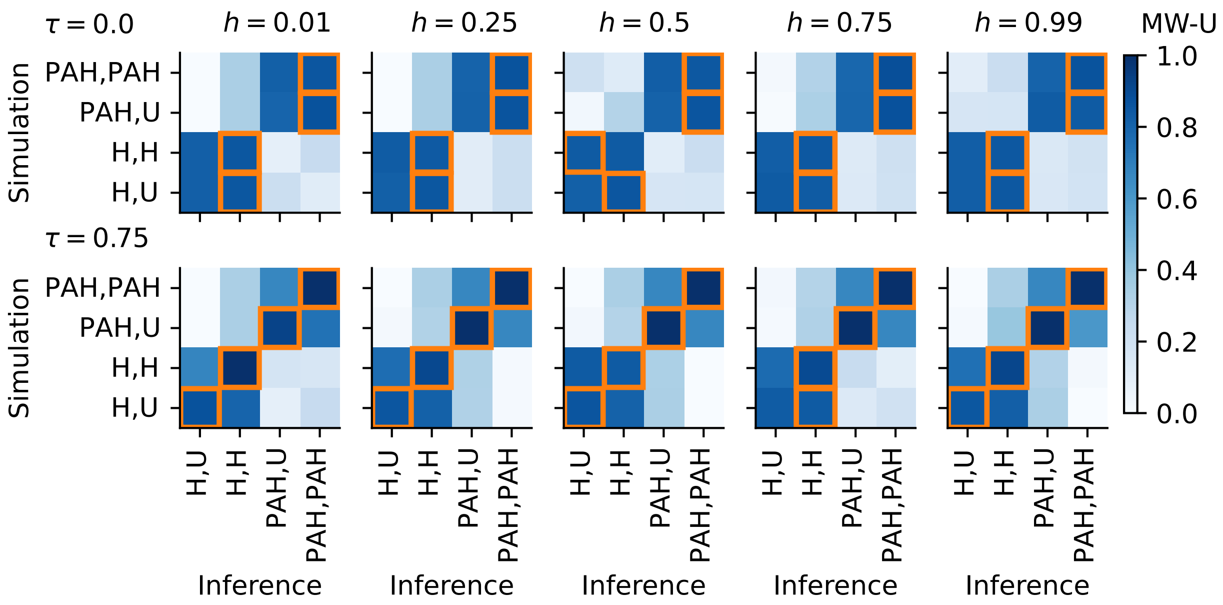

Figure S17. **Confusion matrix for model selection**. We simulate networks based on varying homophily h (columns), triadic closure τ (rows) parameters, and link formation mechanisms L_G and L_T (matrix rows). We then fit the model to the simulated data and plot the Mann-Whitney test statistic (MW-U) comparing the sampled distances to the unified distribution of all other model variants' distance samples. Higher values indicate smaller distances compared to the other models, that is, a better fit. We then select the model with the highest MW-U value for each true model (column-based selection, marked by orange squares). A perfect fit would be indicated by a diagonal line of orange squares. For $\tau = 0$ (upper row), the selection confuses variants with identical global selection mechanisms L_G but different triadic closure mechanisms L_T which is expected due to the lack of triadic closure. For $\tau = 0.75$ (lower row), the selection is more accurate. It only confuses the true model (H, U) with (H, H), under moderate homophily $h = 0.75$.

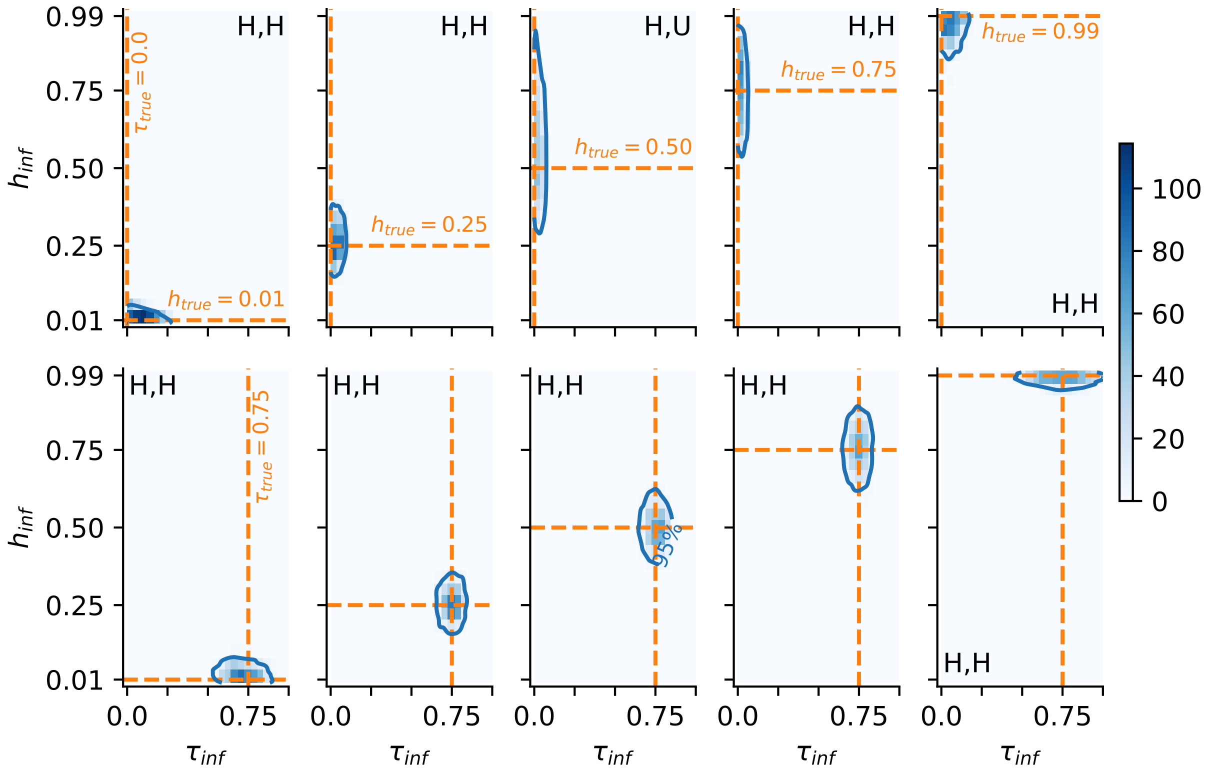

Figure S18. (H, H) **posterior distributions inference**. We simulate (H, H) networks based on varying homophily h_{true} and triadic closure τ_{true} parameters (orange lines) and infer the posterior distributions of the homophily h_{inf} and triadic closure τ_{inf} parameters (blue heatmap) using the best fitting model variant (model labels in corners). 95% of the probability mass is contained in the area marked by the blue contours. Ideally, the posterior distribution should be centered closely around the intersecting true parameters (orange lines).

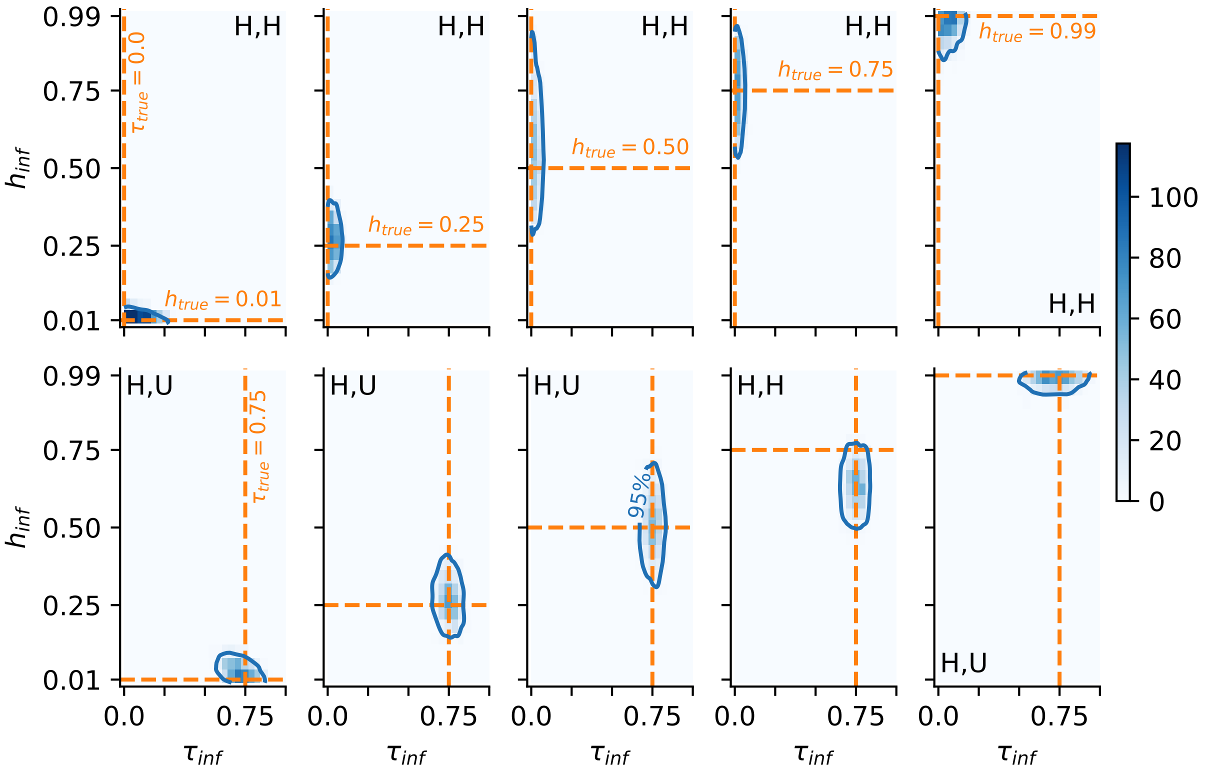

Figure S19. (H, U) **posterior distributions inference**. We simulate (H, U) networks based on varying homophily h_{true} and triadic closure τ_{true} parameters (orange lines) and infer the posterior distributions of the homophily h_{inf} and triadic closure τ_{inf} parameters (blue heatmap) using the best fitting model variant (model labels in corners). 95% of the probability mass is contained in the area marked by the blue contours. Ideally, the posterior distribution should be centered closely around the intersecting true parameters (orange lines).

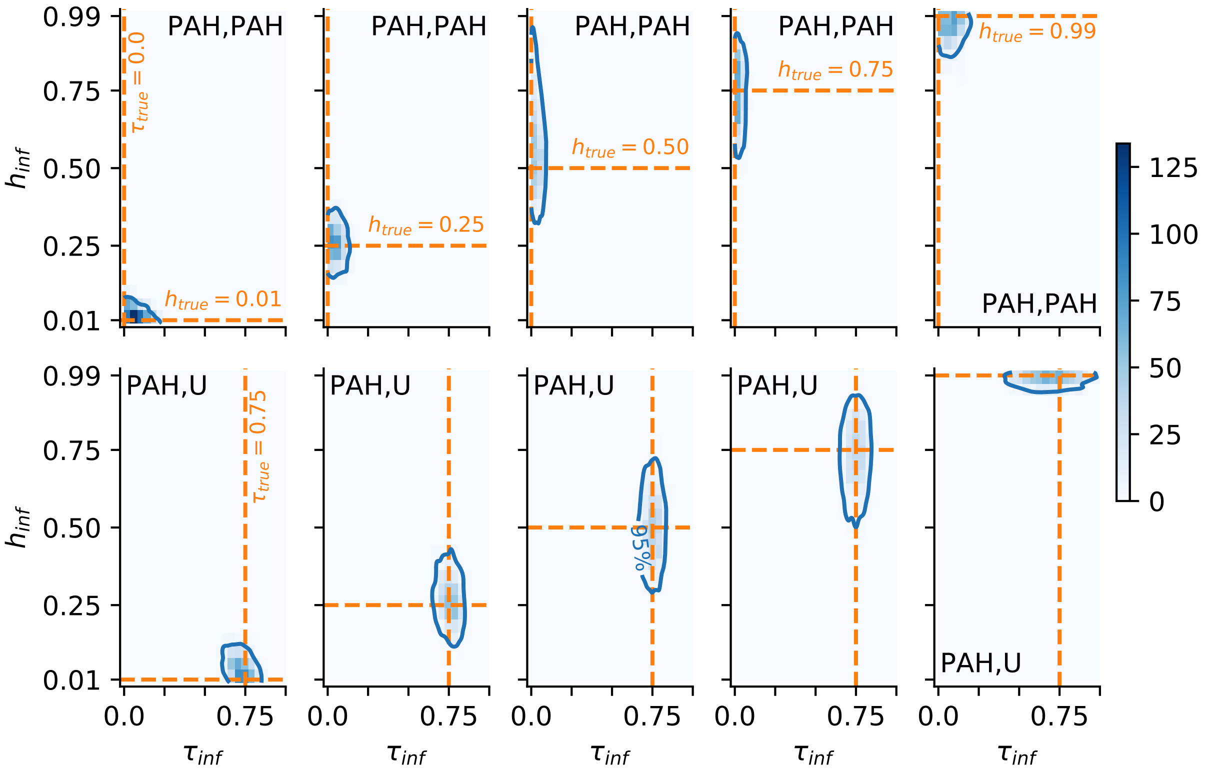

Figure S20. $(\mathrm{PAH}, \mathrm{U})$ **posterior distributions inference**. We simulate $(\mathrm{PAH}, \mathrm{U})$ networks based on varying homophily h_{true} and triadic closure τ_{true} parameters (orange lines) and infer the posterior distributions of the homophily h_{inf} and triadic closure τ_{inf} parameters (blue heatmap) using the best fitting model variant (model labels in corners). 95% of the probability mass is contained in the area marked by the blue contours. Ideally, the posterior distribution should be centered closely around the intersecting true parameters (orange lines).

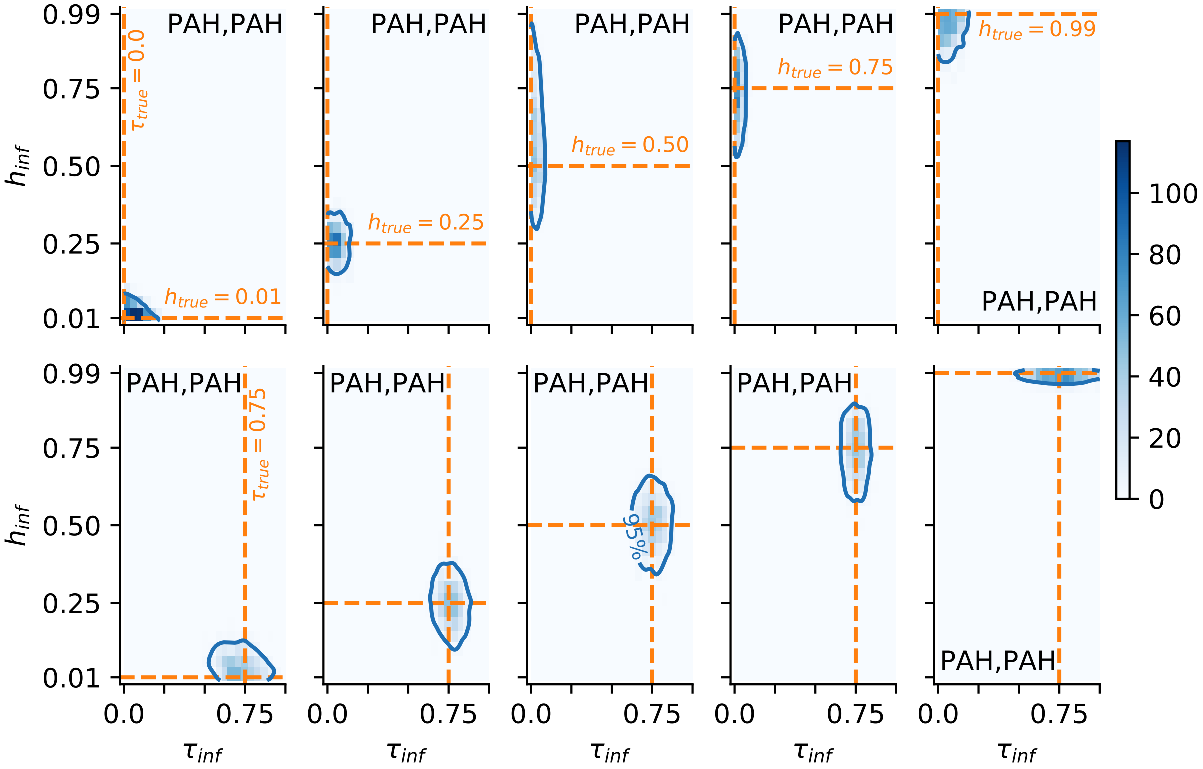

Figure S21. (PAH, PAH) **posterior distributions inference**. We simulate (PAH, U) networks based on varying homophily h_{true} and triadic closure τ_{true} parameters (orange lines) and infer the posterior distributions of the homophily h_{inf} and triadic closure τ_{inf} parameters (blue heatmap) using the best fitting model variant (model labels in corners). 95% of the probability mass is contained in the area marked by the blue contours. Ideally, the posterior distribution should be centered closely around the intersecting true parameters (orange lines).

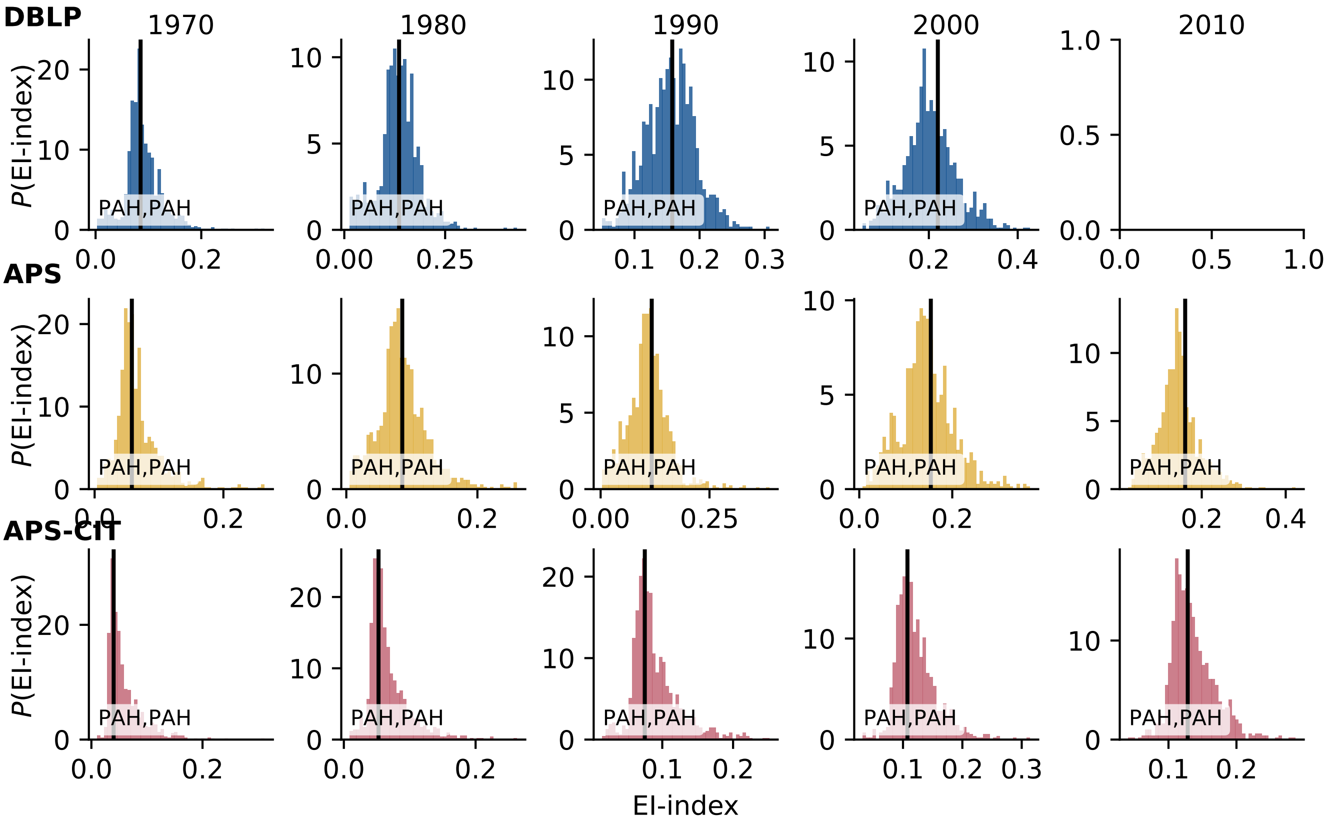

Figure S22. **EI-index predictive analysis**. For all h and τ approximate posterior sample pairs, we compute the EI-index average of 100 PATCH simulations. We compare the histogram over all 1,000 pairs to the observed EI-index (vertical line) to see if PATCH can reproduce it. Network segregation is well captured by PATCH in all datasets and decades.

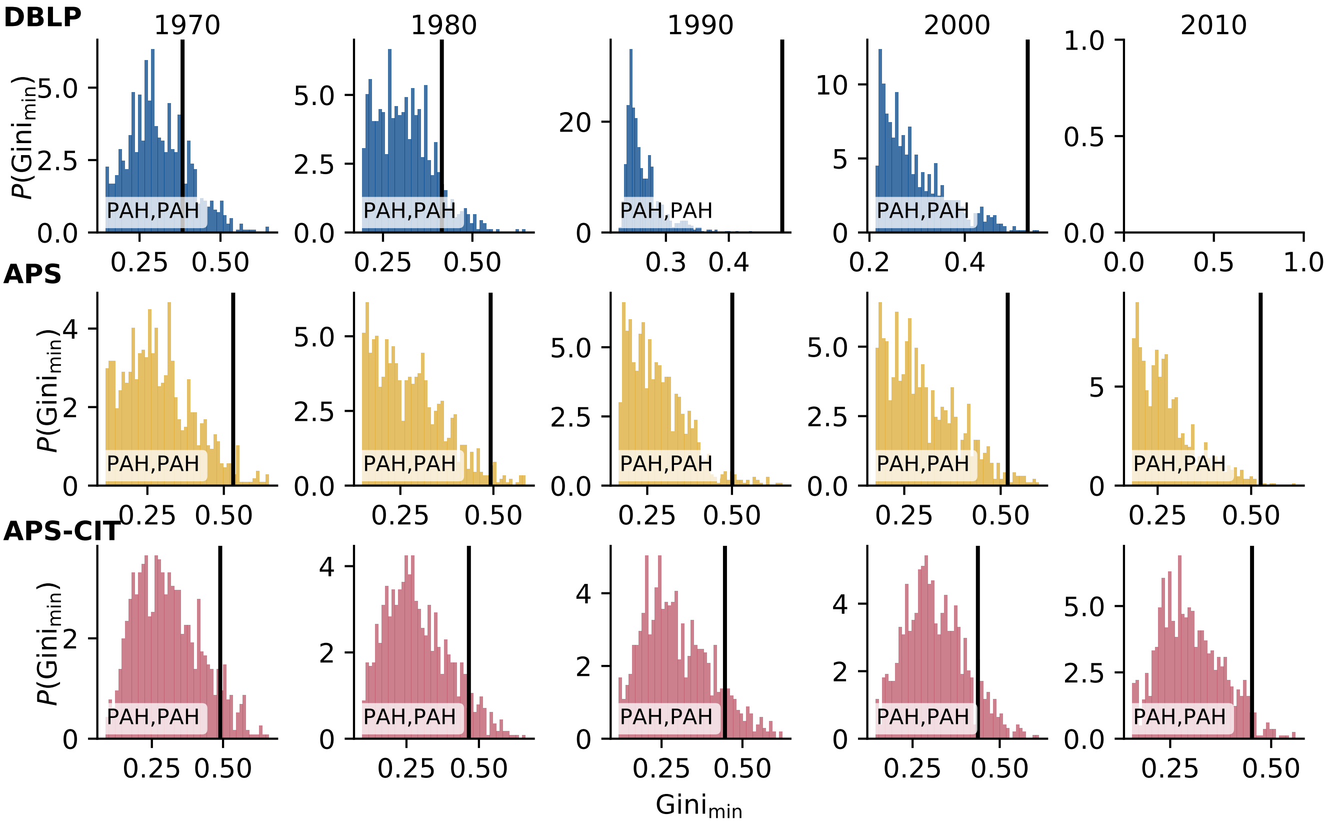

Figure S23. $\mathrm{Gini}_{\mathrm{min}}$ **predictive analysis**. For all h and τ approximate posterior sample pairs, we compute the $\mathrm{Gini}_{\mathrm{min}}$ average of 100 PATCH simulations. We compare the histogram over all 1,000 pairs to the observed $\mathrm{Gini}_{\mathrm{min}}$ (vertical line) to see if PATCH can reproduce it. PATCH cannot reproduce the degree inequality among minority nodes observed in all networks.

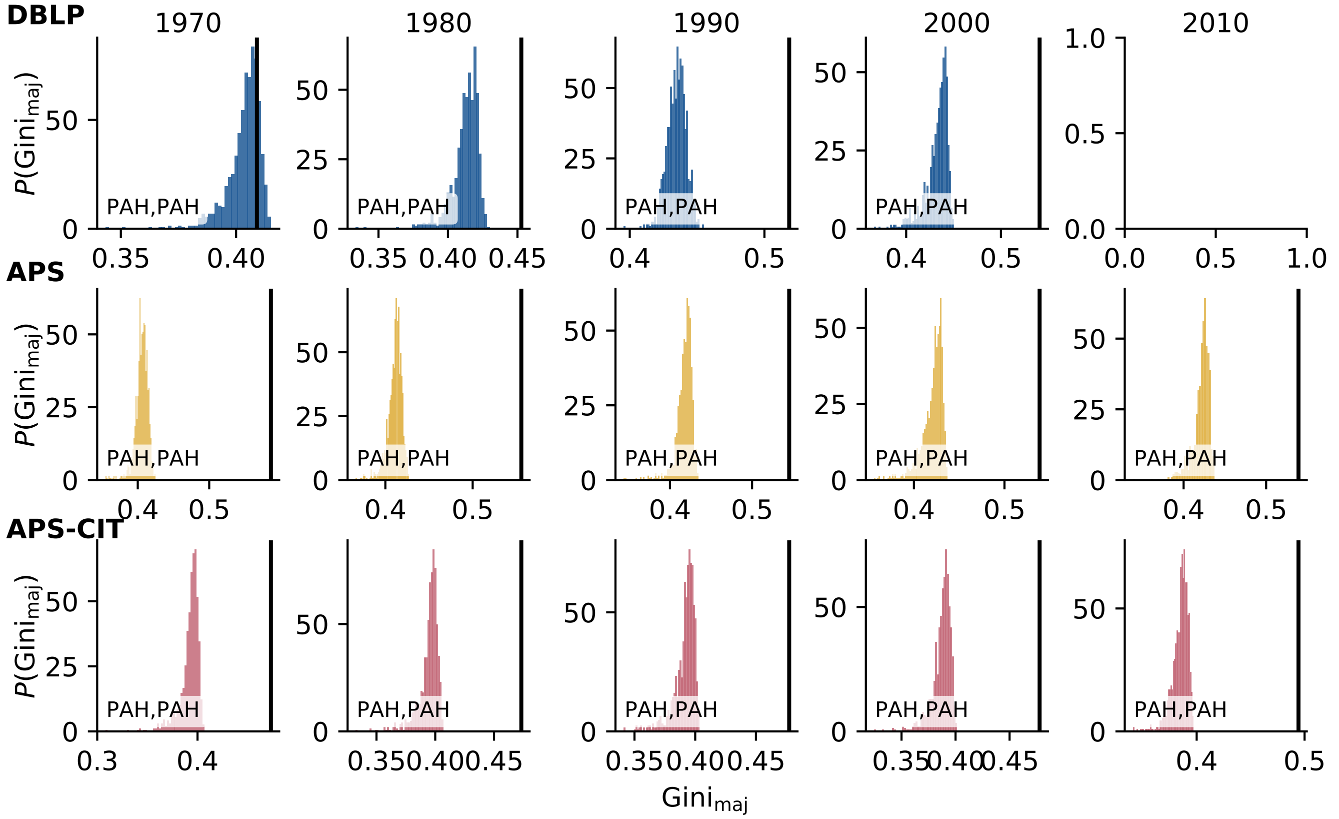

Figure S24. Gini_{maj} **predictive analysis**. For all h and τ approximate posterior sample pairs, we compute the Gini_{maj} average of 100 PATCH simulations. We compare the histogram over all 1,000 pairs to the observed Gini_{maj} (vertical line) to see if PATCH can reproduce it. PATCH underestimates the degree inequality among majority nodes consistently.

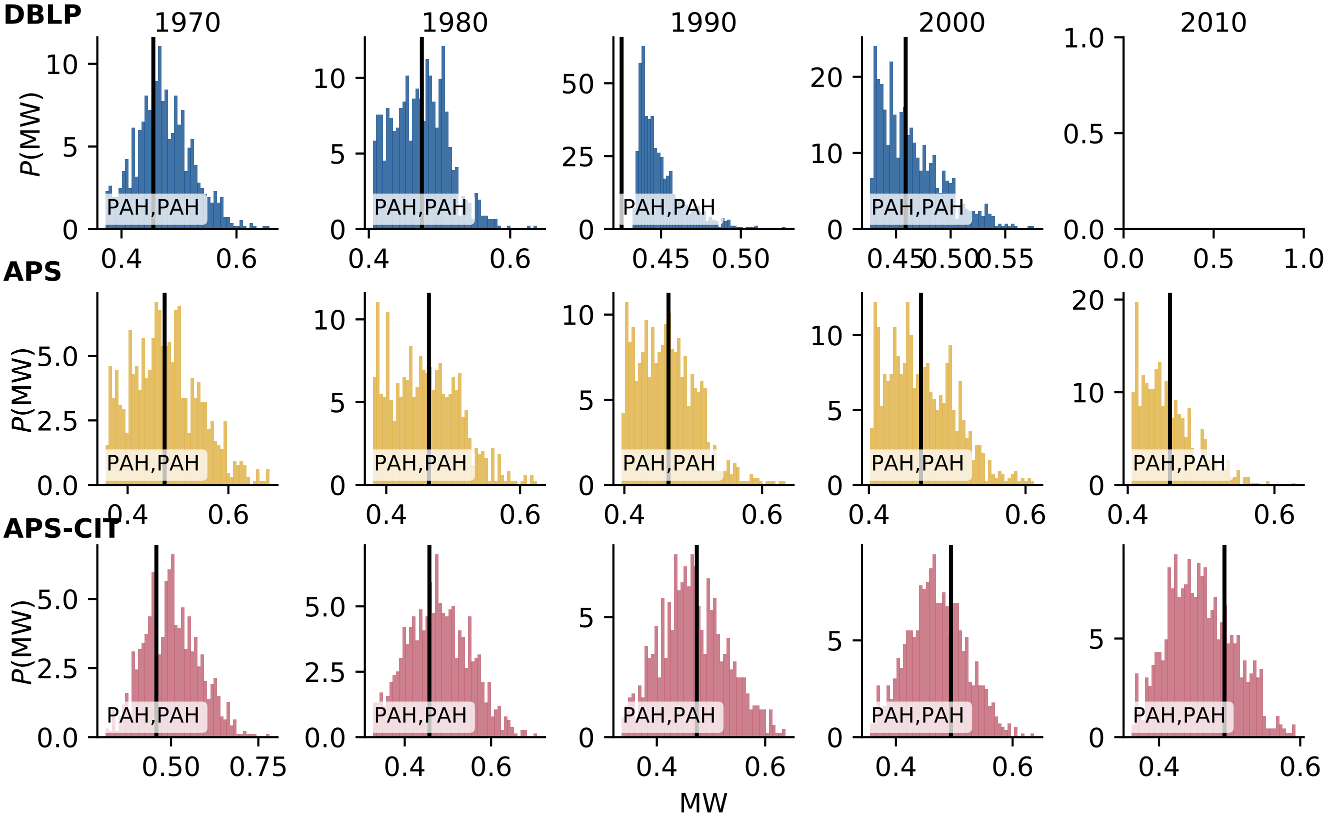

Figure S25. MW **predictive analysis**. For all h and τ approximate posterior sample pairs, we compute the Mann-Whitney (MW) test statistic average of 100 PATCH simulations. We compare the histogram over all 1,000 pairs to the observed MW (vertical line) to see if PATCH can reproduce it. PATCH reproduces the between-group degree inequality observed in most networks.

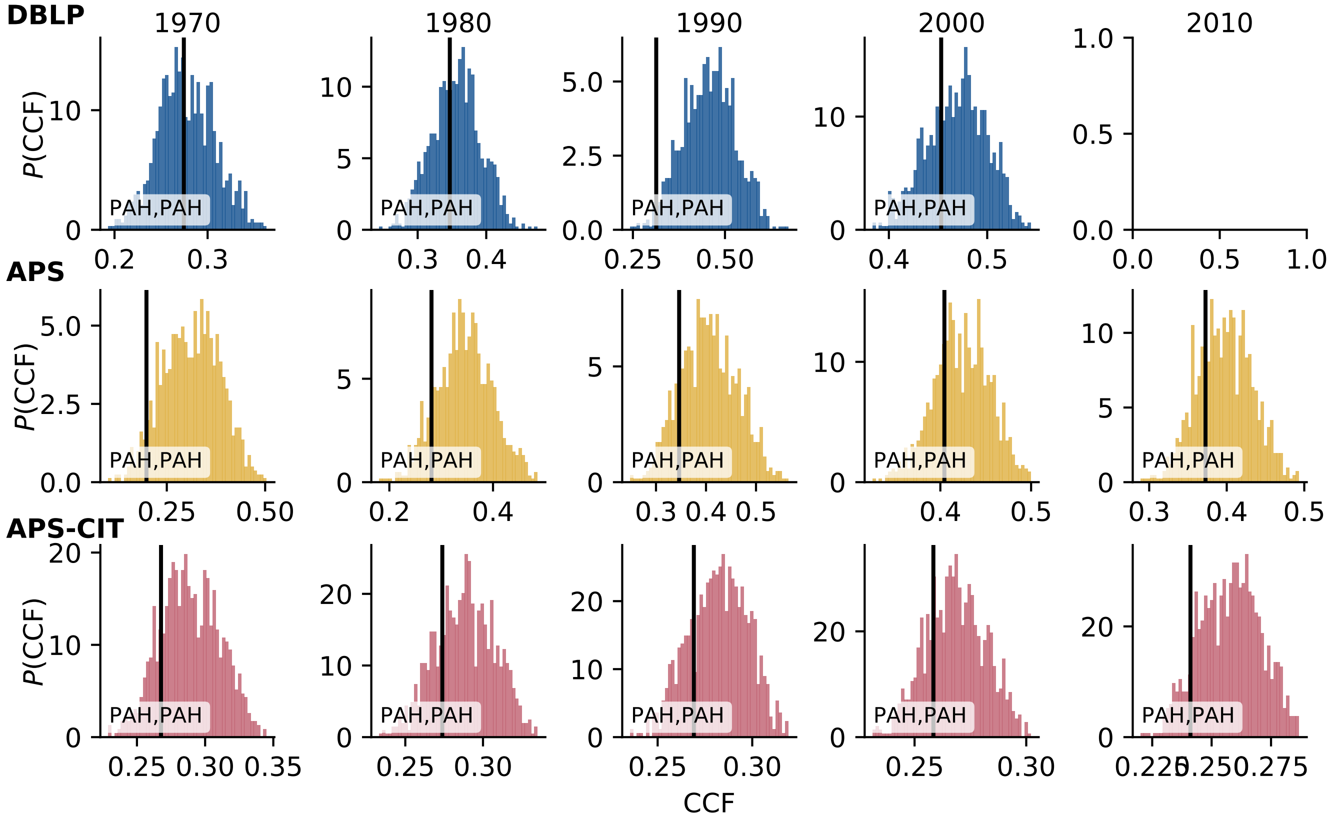

Figure S26. CCF **predictive analysis**. For all h and τ approximate posterior sample pairs, we compute the clustering coefficient (CCF) average of 100 PATCH simulations. We compare the histogram over all 1,000 pairs to the observed CCF (vertical line) to see if PATCH can reproduce it. PATCH reproduces the observed network clustering in most cases.

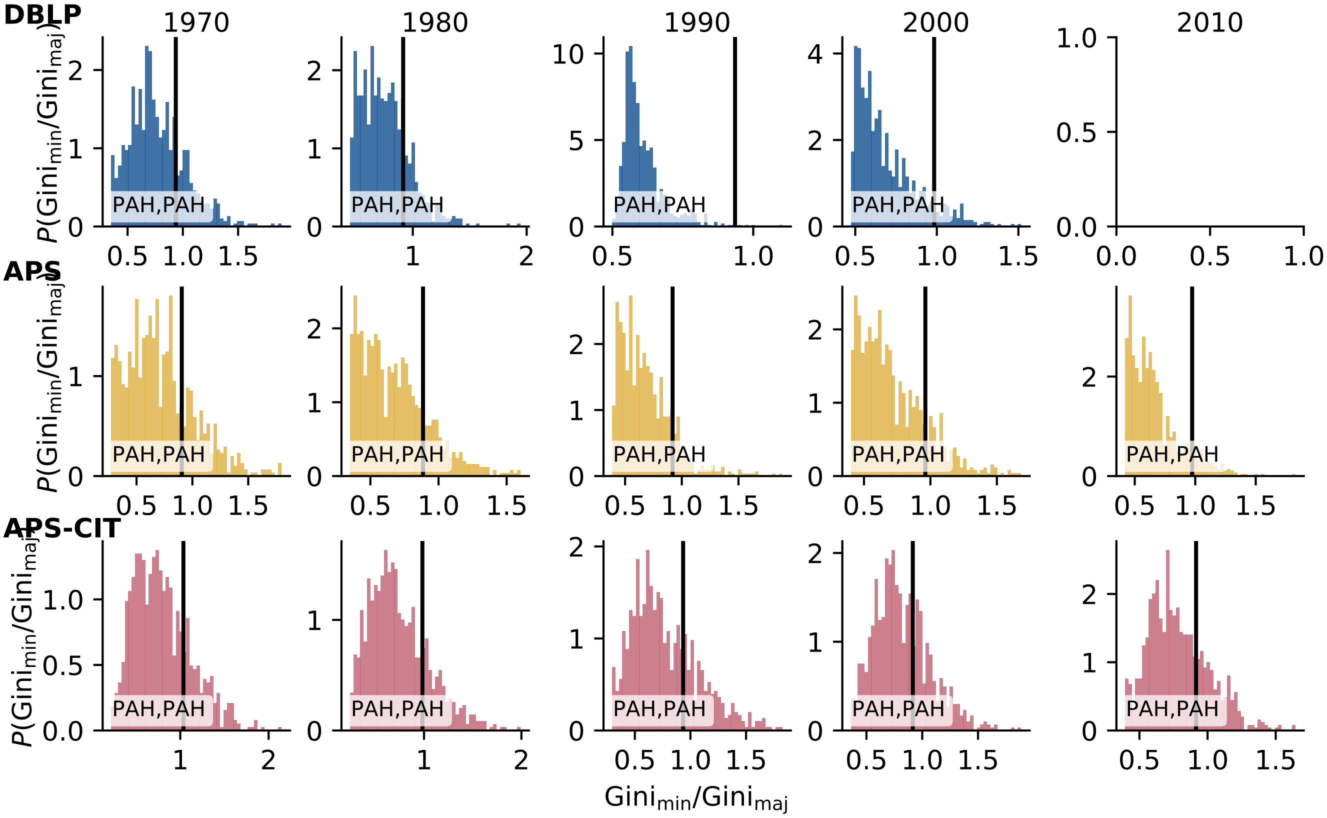

Figure S27. $\text{Gini}_{\text{min}}/\text{Gini}_{\text{maj}}$ **predictive analysis**. For all h and τ approximate posterior sample pairs, we compute the $\text{Gini}_{\text{min}}/\text{Gini}_{\text{maj}}$ average of 100 PATCH simulations. We compare the histogram over all 1,000 pairs to the observed $\text{Gini}_{\text{min}}/\text{Gini}_{\text{maj}}$ (vertical line) to see if PATCH can reproduce it. PATCH roughly matches the within-group inequality ratio although it is not used during the inference. However, it consistently assigns a stronger inequality to the majority group.
